# Supplementary material for: Conformational changes and CO2-induced channel gating in connexin26
Source: Structure. 2022 May 5;30(5):697–706.e4. doi: 10.1016/j.str.2022.02.010 (PMC9592558; doi:10.1016/j.str.2022.02.010)
Supplement: Document S1. Figures S1–S11 and Tables S1–S3 [file mmc1.pdf]

Structure, Volume *30*

Supplemental Information

Conformational changes and CO_2-induced channel gating in connexin26

Deborah H. Brotherton, Christos G. Savva, Timothy J. Ragan, Nicholas Dale, and Alexander D. Cameron

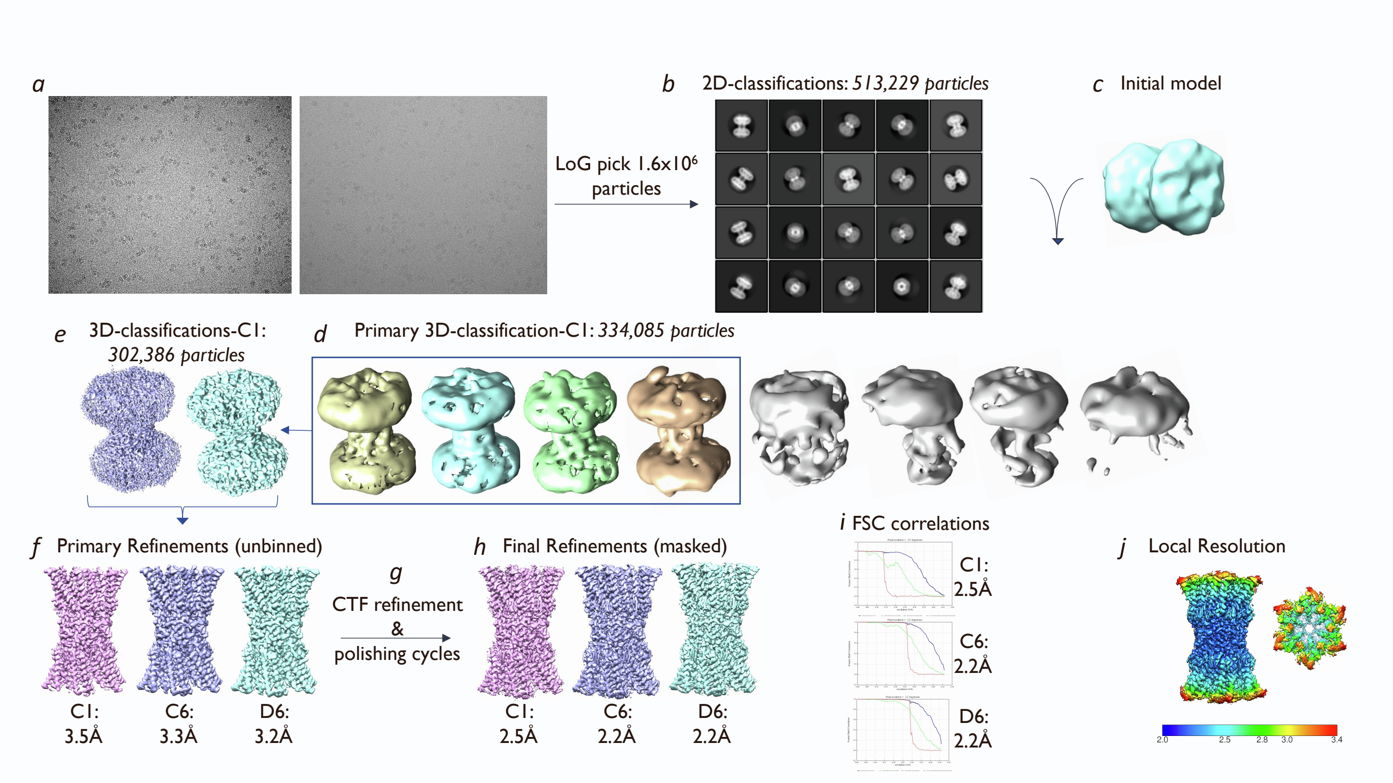

Figure S1: Workflow for processing of cryo-EM data associated with 55mmHg PCO_2 Related to Star Methods. The resolution of reconstructions using C1, C6 and D6 symmetry is shown before and after multiple rounds of particle polishing and CTF refinement. The local resolution map is coloured according to resolution estimated in ResMap (Kucukelbir et al., 2014).

a

Buffer	αCSF20	αCSF55	αCSF90
%CO_2	2.5	10	15
pH pre-gas	7.9	8.7	8.8
pH	7.5	7.5	7.4

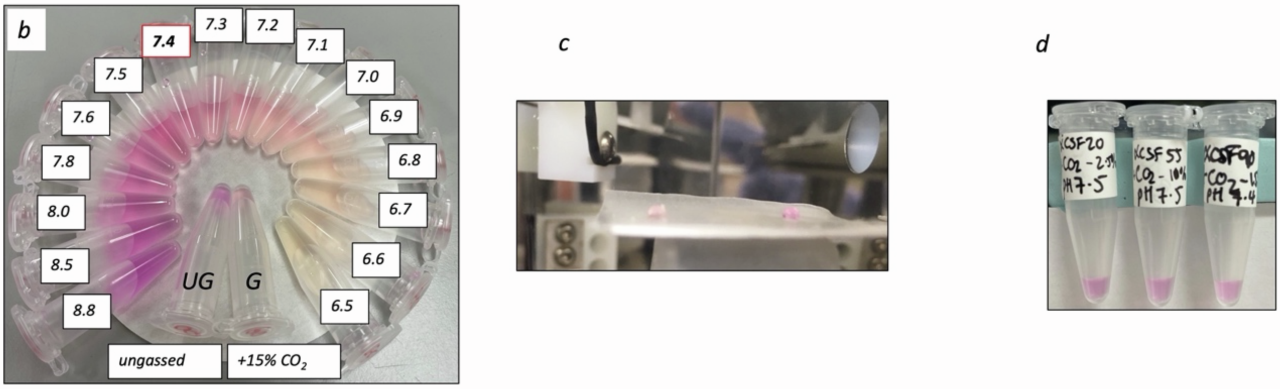

Figure S2: **Control of pH of samples before vitrification.** Related to Star Methods. Controls were carried out to ensure that the pH of the samples at vitrification was the same irrespective of the PCO_2 level. Gassing of the sample with CO_2 is required to obtain the necessary level of PCO_2. **a)** 60 μl samples of each buffer before and after gassing with the indicated percentage of CO_2 showing that all three buffers have the same pH after gassing. The pH was measured using an ultra-microprobe pH meter. **b-d)** To ensure that the pH of the protein remained constant during the blotting procedure samples were tested using Phenol Red as a pH indicator. **b)** To obtain a colour reference for the pH indicator dye, 2μl of Phenol Red was added to 400μl samples of the 90mmHg buffer buffer that had been adjusted to the required pH. The samples prepared as in (a) with 15% CO_2 are shown at the bottom of the wheel (UG not gassed, G gassed). **c)** 3 μl drops of buffer gassed as for protein preparation pre-vitrification (left) and ungassed buffer (right) in the plunge freezer chamber after 30 seconds in the CO_2 - equilibrated atmosphere as described in the methods. The respective colours of the drops do not change over the 30-second period indicating that the pH remains stable during this time. 30 seconds was chosen as being representative of the maximum time taken between applying sample to the grid, and the grid entering the liquid ethane. **d)** The three gassed buffers from (a) with Phenol Red have the same colour.

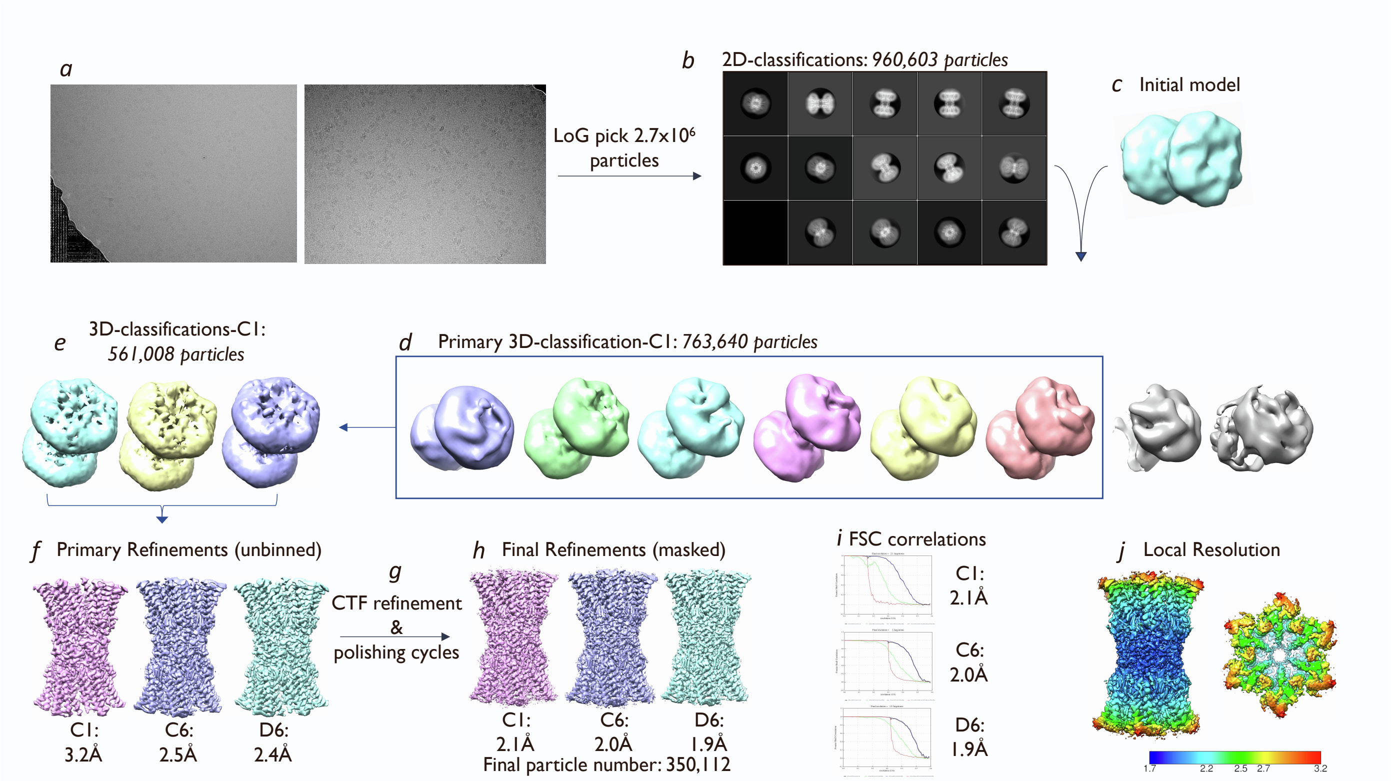

Figure S3: Workflow for processing of cryo-EM data associated with 90mmHg PCO$_2$. Related to Star Methods. As for Fig. S1 the resolution of reconstructions using C1, C6 and D6 symmetry is shown before and after multiple rounds of particle polishing and CTF refinement. The local resolution map is coloured according to resolution estimated in ResMap (Kucukelbir et al., 2014).

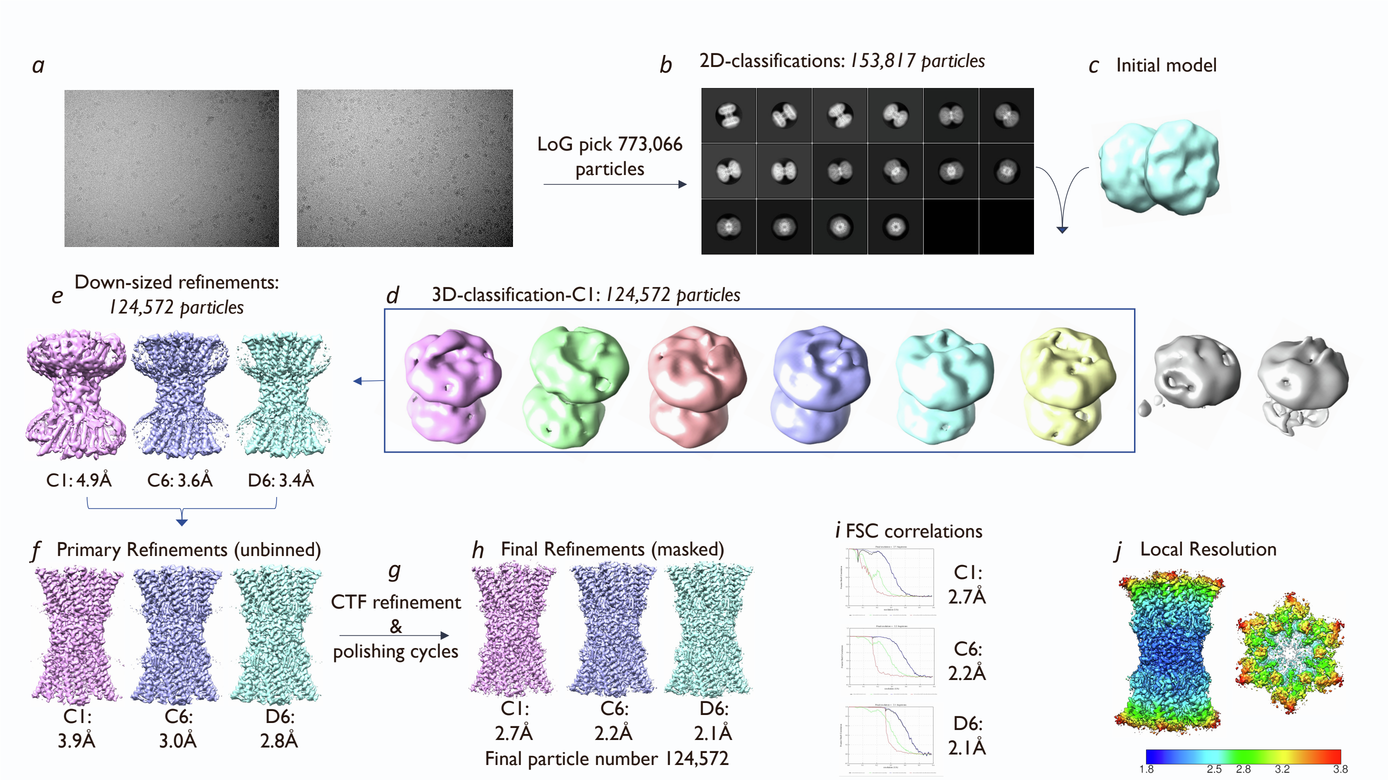

Figure S4: Workflow for processing of cryo-EM data associated with 20mmHg PCO_2. Related to Star Methods. As for Fig. S1 the resolution of reconstructions using C1, C6 and D6 symmetry is shown before and after multiple rounds of particle polishing and CTF refinement. The local resolution map is coloured according to resolution estimated in ResMap (Kucukelbir et al., 2014).

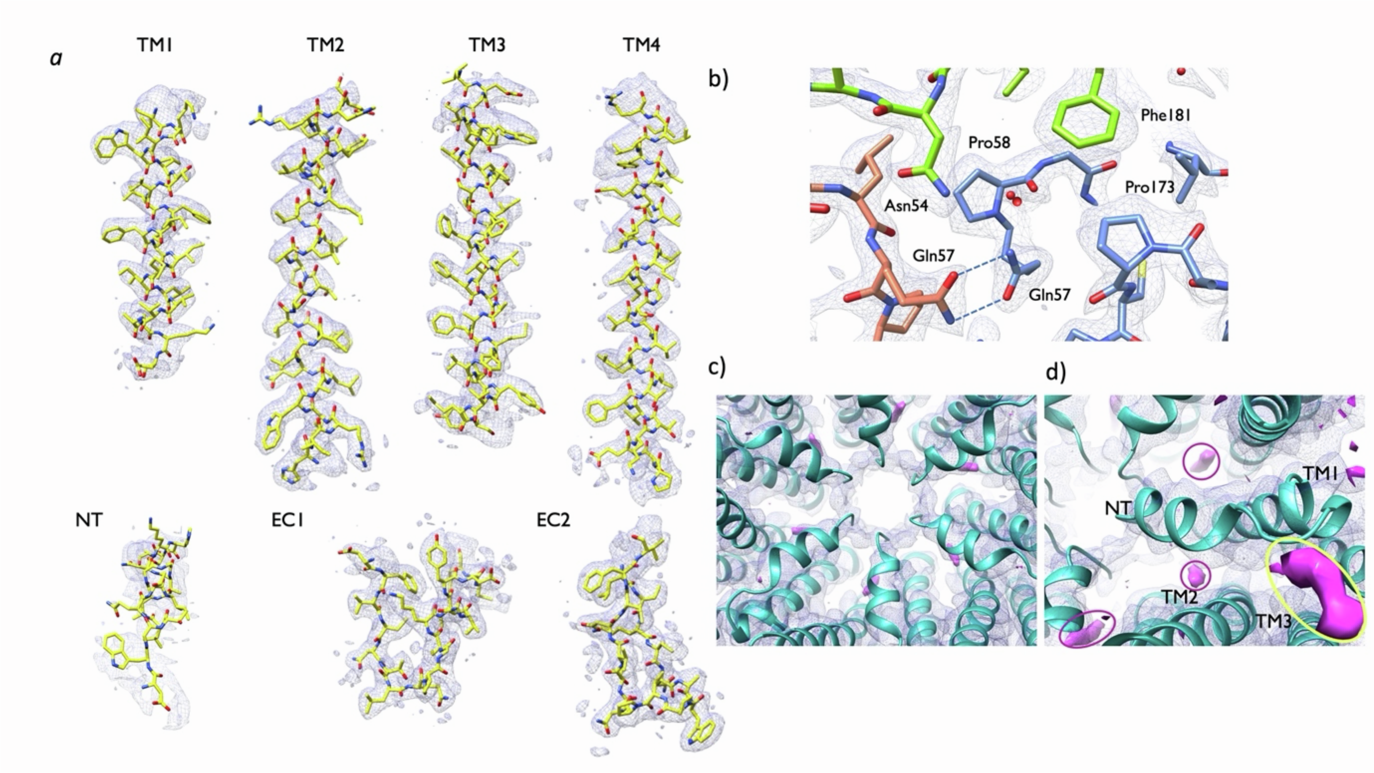

Figure S5: Density for 55 mmHg PCO_2 maps. Related to Star Methods. **a)** Density associated with main structural features (Transmembrane (TM) helices 1-4, the N-terminal helix (NT) and Extracellular (EC) loops 1 and 2.) No sharpening has been applied. (The N-terminus has been shown with a lower threshold (0.0117 *vs* 0.0126).) **b)** Density associated with the gap junction with a map sharpened in Relion (Sharpening B - 22Å^2). **c)** The density associated with the N-terminus can be seen to form a ring in the centre of the pore. **d)** Sausage-shaped densities were observed within the pore (solid magenta circled in magenta) as was density extending from TM3 to the N-terminal helix (solid magenta circled in yellow).

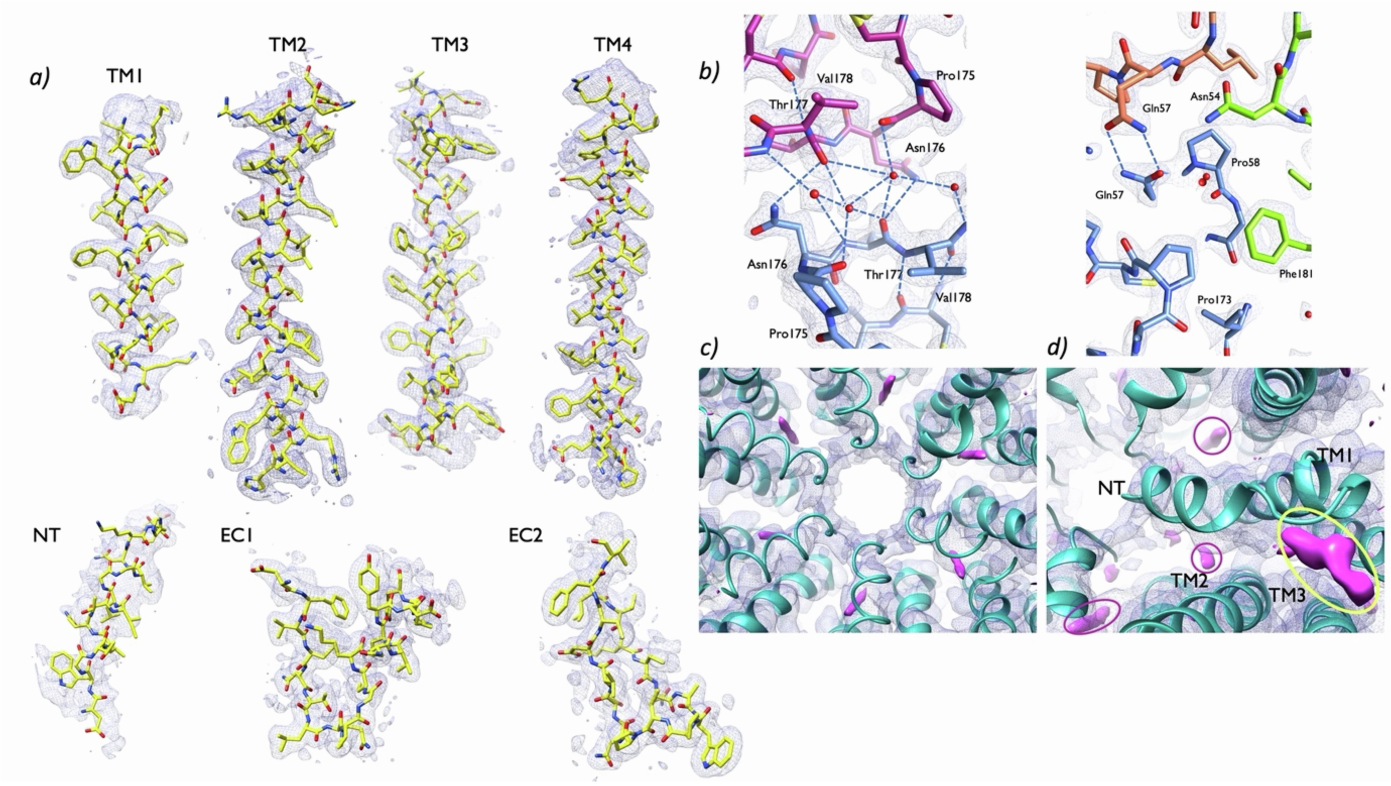

Figure S6: Density for 90mmHg PCO_2 maps. Related to Star Methods. Equivalent to Fig. S2 **a)** Density associated with main structural features (Transmembrane (TM) helices 1-4, the N-terminal helix (NT) and Extracellular (EC) loops 1 and 2.) No sharpening has been applied. (The N-terminus has been shown with a lower threshold (0.0065 *vs* 0.0069).) **b)** Density associated with the gap junction with a map sharpened in phenix.autosharp (Adams et al., 2010). **c)** The density associated with the N-terminus can be seen to form a ring in the centre of the pore. **d)** Sausage-shaped densities were observed within the pore (solid magenta circled in magenta) as was density extending from TM3 to the N-terminal helix (solid magenta circled in yellow).

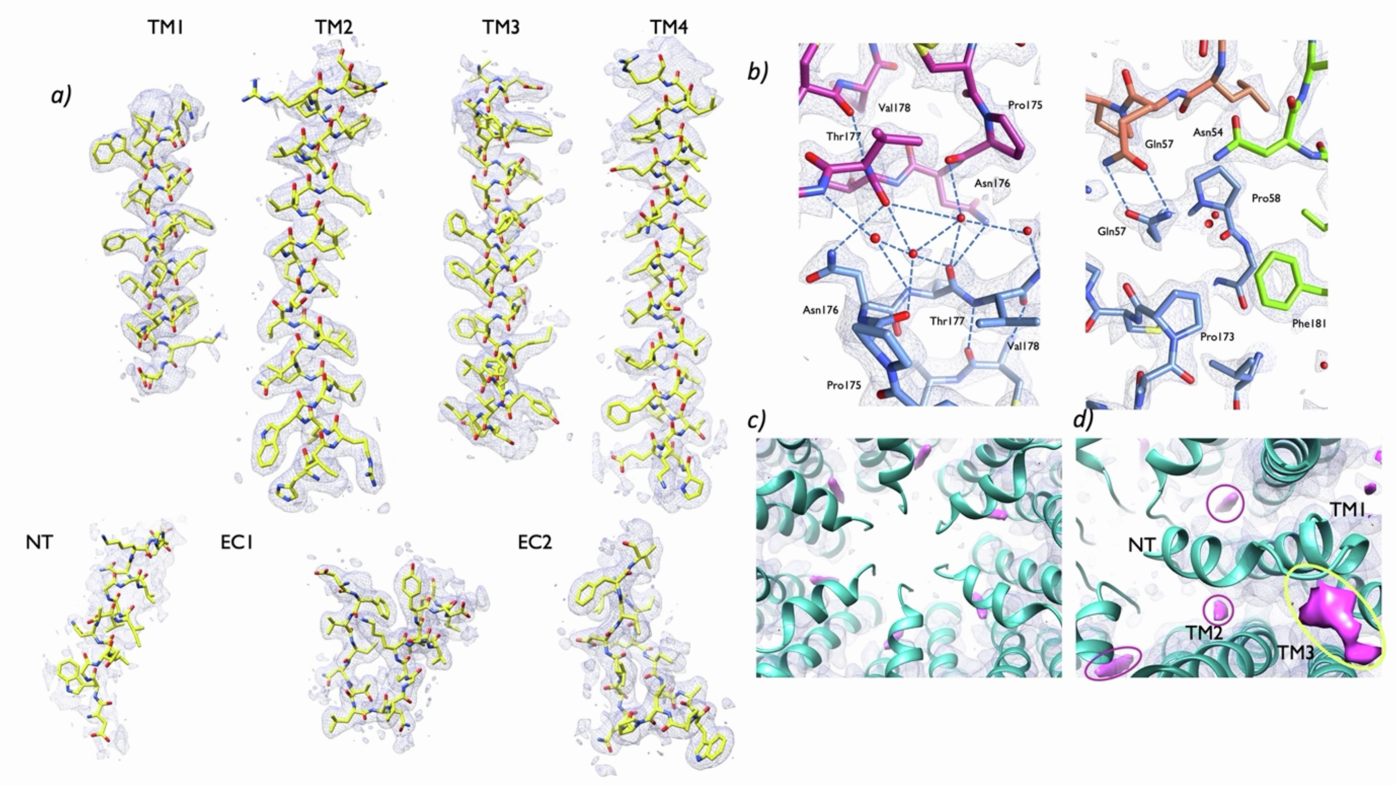

Figure S7: Density for 20mmHg PCO_2 maps. Related to Star Methods. Equivalent to Fig. S2 **a)** Density associated with main structural features (Transmembrane (TM) helices 1-4, the N-terminal helix (NT) and Extracellular (EC) loops 1 and 2.) No sharpening has been applied. (The N-terminus has been shown with a lower threshold (0.0065 *vs* 0.0069).) **b)** Density associated with the gap junction with a map sharpened in phenix.autosharp (Adams et al., 2010). **c)** The density associated with the N-terminus is much less defined than in Figs S5 and S6. **d)** Sausage-shaped densities were observed within the pore (solid magenta circled in magenta) as was density extending from TM3 to the N-terminal helix (solid magenta circled in yellow).

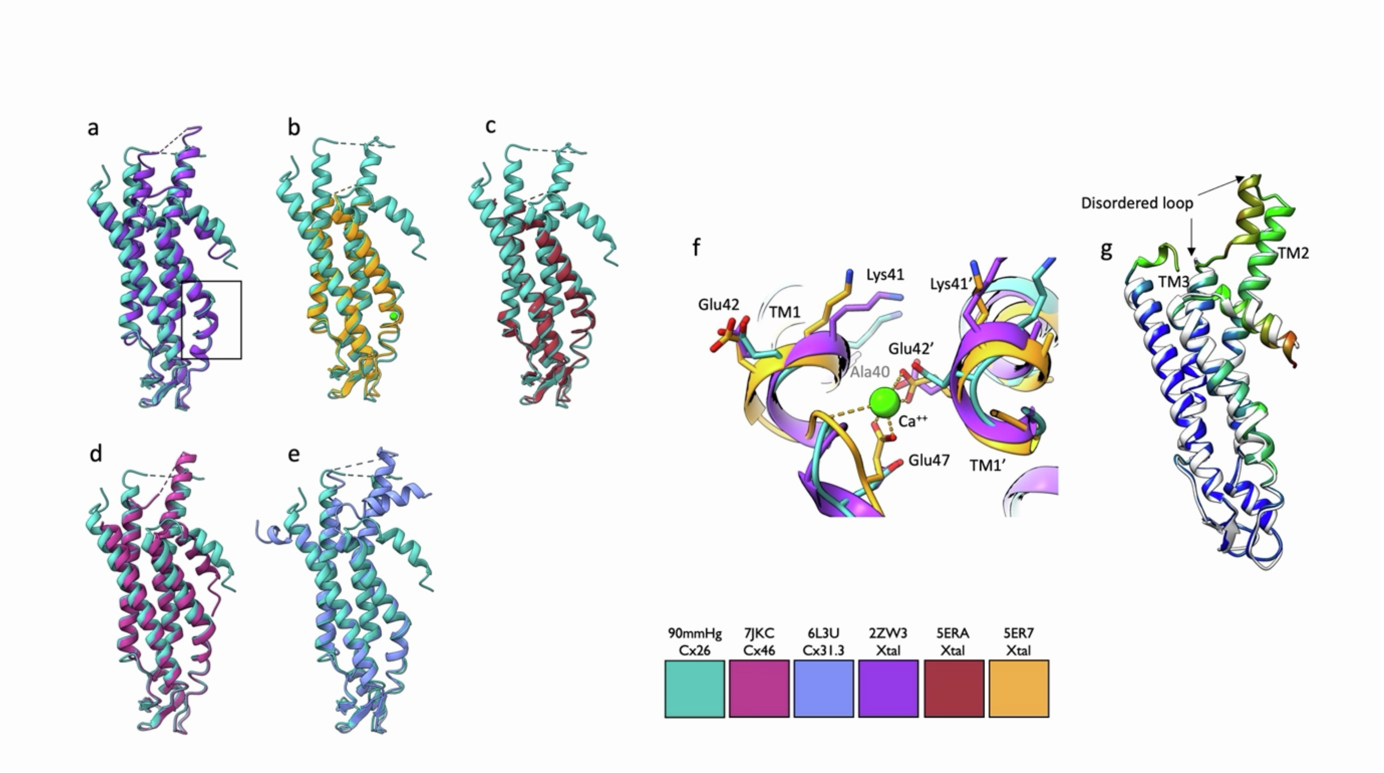

Figure S8: Comparison of structures of connexins. Related to Figure 3. Structure of a subunit of 90mmHg PCO_2 Cx26 cryo-EM structure (sea-green) superposed on: **a)** Cx26 crystallised in UDM (Maeda et al., 2009) (2ZW3, purple); **b)** Cx26 calcium-bound, crystallised in a facial amphiphile (Bennett et al., 2016) (5ER7, orange); **c)** Cx26 crystallised as in (b) but without calcium (5ERA, brown); **d)** cryo-EM structure of Cx46 (Flores et al., 2020) (7JKC, burgundy) and **e)** cryo-EM structure of Cx31.3 (Lee et al., 2020) (6L3U, sky blue). The N-termini are not observed in either 5ER7 or 5ERA and are in a raised position in 6L3U. The region between EC1 and TM1 (boxed in (a), residues Val 37 to Glu 42) varies amongst the Cx26 structures. Using the refined cryo-EM structure as a search model for molecular replacement against structure factor amplitudes associated with either the 5ER7 or 5ERA models clearly shows the resulting density to be consistent with the crystallographic models. **f)** Comparison of the cryo-EM structure with 2ZW3 and 5ER7 with the same colouring scheme as in a-e. Residues between Val37 and Glu42 vary amongst all the structures; residues Glu42 to Glu47 between the calcium-bound and non-bound structures. **g)** Structure of Cx26 cryo-EM structure (90mmHg PCO_2 white) superposed on a model predicted by AlphaFold (Jumper et al., 2021). The model from AlphaFold is coloured by confidence level with low (red) to high (blue) confidence spanning the colours of the rainbow. The boundaries of the loop that is not seen in any of the crystal structures is delimited by arrows. Both AlphaFold and RosettaFold (Baek et al., 2021) consistently predict a similar conformation, though with low confidence levels.

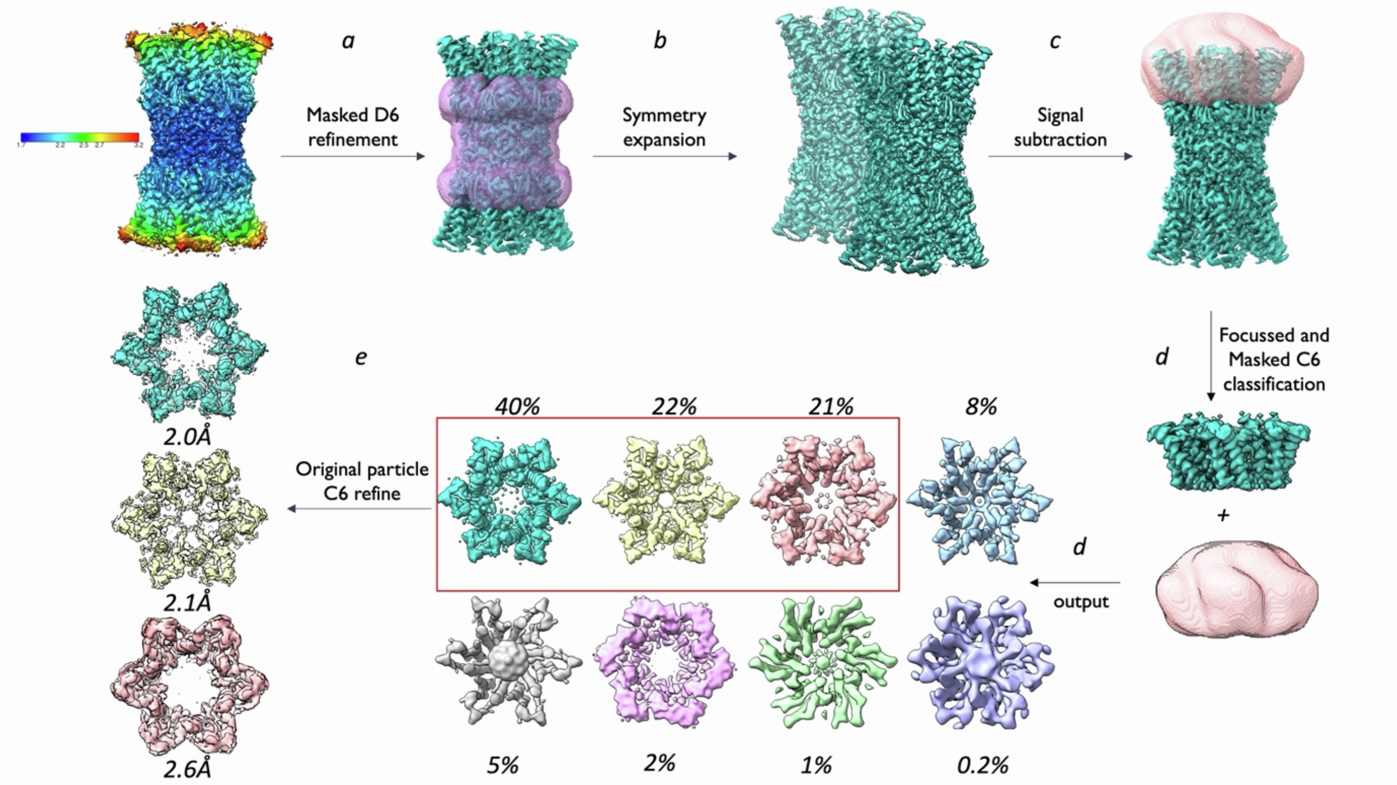

Figure S9: C6 classification of the 90mmHg D6 refined particles. Related to Star Methods. **a)** the best refined D6 particles were taken, masked around the region nearer to the hemichannel docking site, and refined further **b)** these particles were symmetry expanded in D1 **c)** the symmetry expanded particle set were signal subtracted using a second mask covering the least-defined area. **d)** these particles were centred, and classified imposing C6 symmetry, fixed angles, and 8 classes. **e)** the top three classes were selected, and the original particles refined with C6 symmetry imposed, using a mask covering one hemichannel. The colours of the reconstructions after refinement match those in the red box.

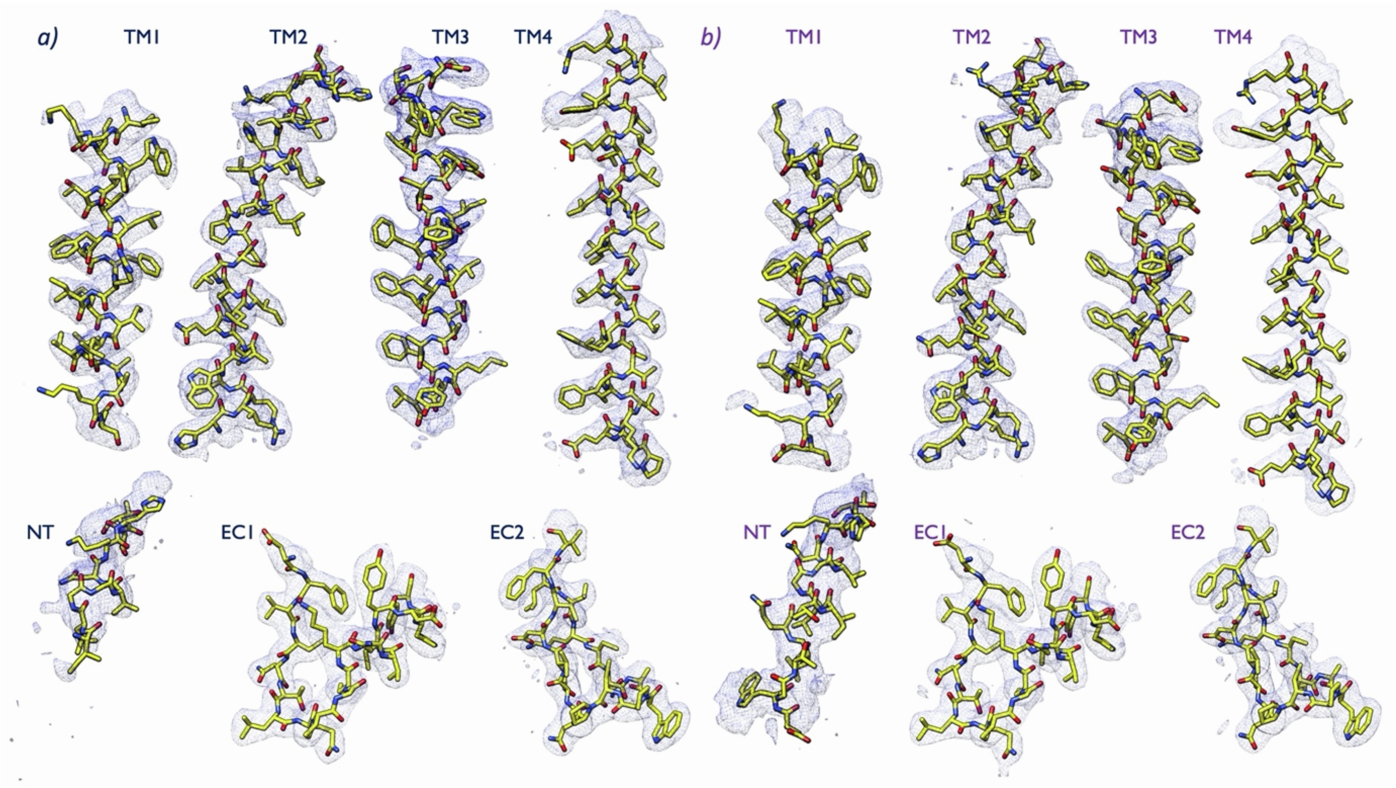

Figure S10: Quality of density in maps resulting from C6 classifications in Fig S9. Related to Star Methods. **a)** Density associated with main structural features for the refined particles in class 1. No sharpening has been applied (threshold (0.0075). **b)** Density associated with main structural features for the refined particles in class 2. No sharpening has been applied (threshold (0.0075)).

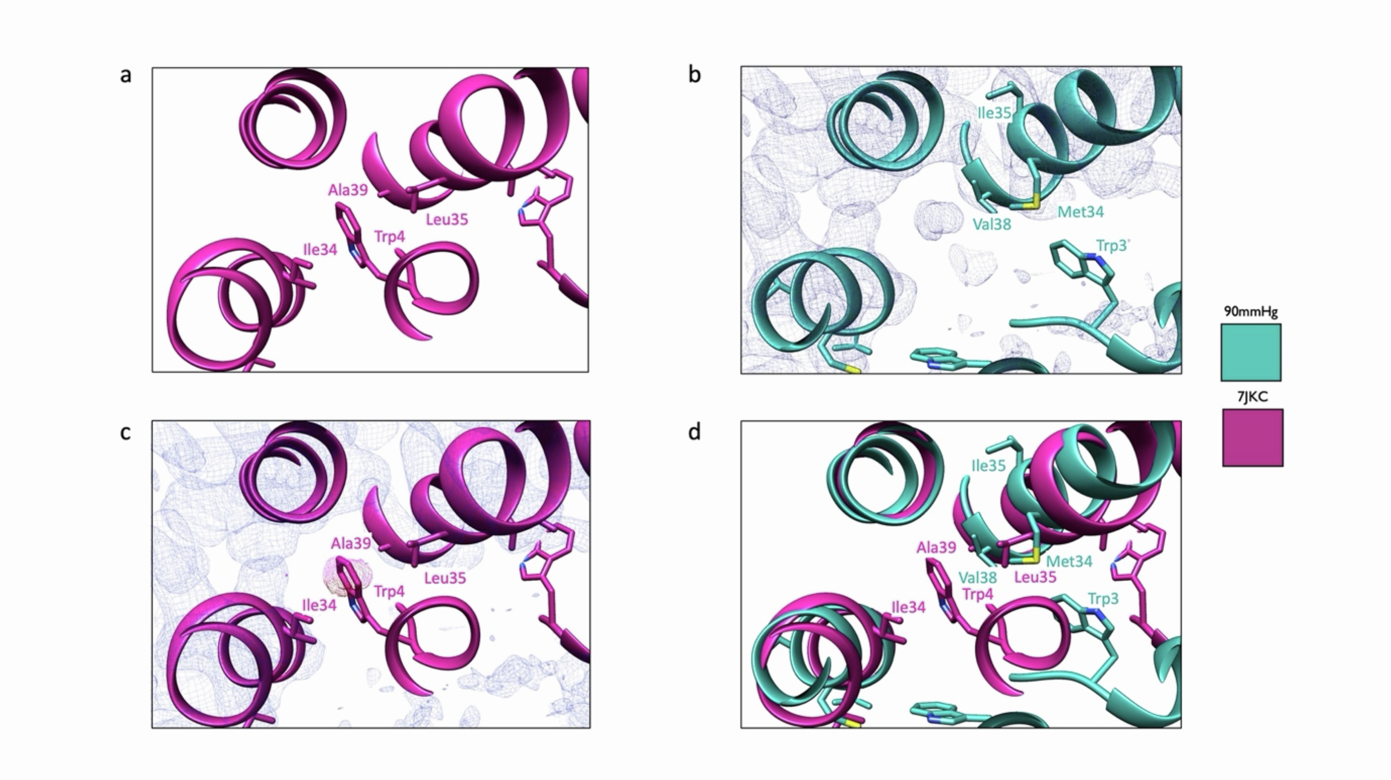

Figure S11: The interaction of the N-terminus with TM1 in Cx26 and Cx46. Related to Star Methods and Figure 3. **a)** Cx46 (7JKC) showing Trp4 seated between Ala39 and Leu35 of TM1 on one subunit and Ile34 on TM1 of the adjoining subunit. **b)** A similar view for the 90mmHg PCO_2 structure with the associated density. **c)** Density as in b) with Cx46 structure as in a) shows the overlap between Trp4 of Cx46 and the lipid like density (pink) associated with the Cx26 structures. **d)** overlay of Cx46 and Cx26, showing the difference in position of the N-terminal Trp.

	55 mmHg CO_2	90 mmHg CO_2	20 mmHg CO_2
Voltage (kV)	300	300	300
Magnification (x1000) X	81	105	105
Camera	K3	K3	K3
Camera mode	Super-resolution	Super-resolution	Super-resolution
Energy filter (eV)	20	20	20
Defocus range (mm)	-2 to -1	-2 to -1	-2 to -1
Pixel size (Å/pix)	1.09	0.85	0.85
Dose on detector (e^-/pix/sec)	10	10	10
Dose on sample (e^-/pix/sec)	10.9	11	11
Exposure time	5	3	3
No. of images	4003	11647	3305
Frames per image	45	45	45
Initial particle number	1679668	2655822	773121
Final particle number	344085	360112	124572
**Resolution**[a]			
masked D6	2.2	1.9	2.1
masked C6	2.2	2.0	2.2
masked C1	2.5	2.1	2.7

a) From Relion_postprocess (Zivanov et al., 2018)

Table S1: Cryo-EM data collection and processing statistics, Related to Star Methods

Refinement	55mm Hg CO_2	90mm Hg CO_2	20mmHg CO_2
Initial model used (PDB code)	2ZW3	55mm Hg CO_2	55mm Hg CO_2
Resolution (Å; FSC=0.5) Map	2.2	1.9	2.1
Sharpening B factor ($Å^2$)	0	0	0
**Model composition**	12 subunits	12 subunits	12 subunits
Non-hydrogen atoms	19097	19313	19205
Protein residues	2244	2268	2256
Water	305	305	305
Ligand: lipid/detergent	60	60	60
**B factor ($Å^2$)**			
protein	71	63	72
water	42	36	42
Lipid/detergent	65	57	64
**R.m.s. deviations**			
Bond lengths (Å)	0.008	0.002	0.004
Bond angles (°)	0.615	0.479	0.501
**Validation**			
MolProbity score	1.45	1.05	1.29
Clashscore	3.04	2.19	1.99
Rotamer outliers (%)	2.94	1.18	2.38
**Ramachandran plot**			
Favoured (%)	98.36	98.92	97.83
Allowed (%)	1.64	1.08	2.17
Disallowed (%)	0.0	0.0	0.0
**Map resolution (Å)**	2.2	1.9	2.1
FSC threshold	0.143	0.143	0.143
Map resolution range (Å) (unmasked)	2.1/2.2/2.2	1.8/1.8/2.0	1.9/1.9/2.2
Map resolution range (Å) (masked)	2.1/2.1/2.2	1.7/1.8/1.9	1.9/1.9/2.1
**Correlation coefficients**			
CC (mask)	0.88	0.90	0.90
CC (box)	0.84	0.82	0.82
CC (peaks)	0.76	0.78	0.76
CC (volume)	0.87	0.89	0.89
Mean CC for ligands	0.80	0.85	0.80

Table S2: Cryo-EM refinement and validation statistics, Related to Star Methods and Figure 1

	**Class 1**	**Class 2**
Initial model used	90mmHg CO_2	90mmHg CO_2
Resolution (Å)	2.0	2.1
Sharpening B factor (Å^2)	0	0
Model composition	6 subunits	6 subunits
Non-hydrogen atoms	9483	9756
Protein residues	1092	1134
Water	297	234
Ligand: lipid/detergent	30	30
**B factor (Å^2)**		
protein	64	73
water	46	48
Lipid/detergent	62	68
**R.m.s. deviations**		
Bond lengths (Å)	0.011	0.011
Bond angles (°)	0.825	0.864
**Validation**		
MolProbity score	2.33	2.65
Clashscore	5.68	8.31
Rotamer outliers (%)	8.59	13.71
**Ramachandran plot**		
Favoured (%)	95.51	95.14
Allowed (%)	3.93	4.32
Disallowed (%)	0.56	0.54
**Map resolution (Å)**	2.1	2.2
FSC threshold	0.143	0.143
Map resolution range (Å) (unmasked)	1.8/1.8/2.0	1.9/2.0/2.2
Map resolution range (Å) (masked)	1.7/1.8/2.0	1.9/1.9/2.1
**Correlation coefficients**		
CC (mask)	0.91	0.9
CC (box)	0.78	0.79
CC (peaks)	0.72	0.72
CC (volume)	0.89	0.89

Table S3: Refinement and Validation Statistics 90mmHg PCO_2 hemichannel models, Related to Star Methods and Figure 4.
