# Supplementary material for: Conformational changes and CO2-induced channel gating in connexin26
Source: Structure. 2022 May 5;30(5):697–706.e4. doi: 10.1016/j.str.2022.02.010 (PMC9592558; doi:10.1016/j.str.2022.02.010)
Supplement: Document S2. Article plus supplemental information [file mmc7.pdf]

Structure

Article

Conformational changes and CO_2-induced channel gating in connexin26

Graphical abstract

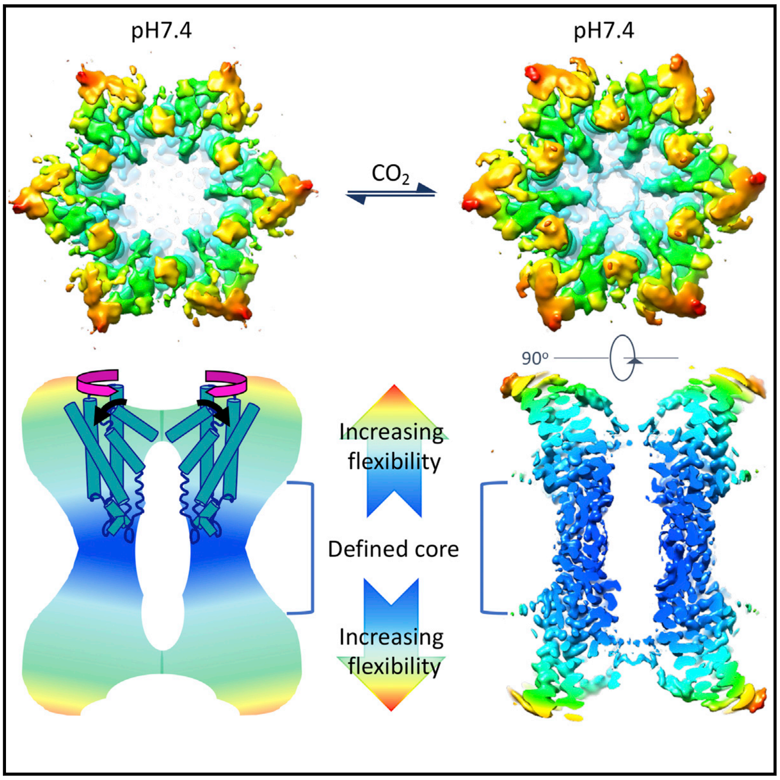

Highlights

- High-resolution cryo-EM structures of connexin26 at varying levels of PCO_2
- CO_2 alone causes conformational changes in the protein under stable pH conditions
- The N-terminal helices regulate the aperture of the pore
- KID syndrome mutations affecting CO_2 sensitivity map to flexion points of structure

Authors

Deborah H. Brotherton, Christos G. Savva, Timothy J. Ragan, Nicholas Dale, Alexander D. Cameron

Correspondence

a.cameron@warwick.ac.uk (A.D.C.), n.e.dale@warwick.ac.uk (N.D.)

In brief

Brotherton et al. present cryo-EM structures of the connexin26 gap junction at three concentrations of CO_2. They show that at constant pH, the aperture of the pore varies depending on CO_2 concentration. Their results correlate with physiological measurements showing that elevated CO_2 closes gap junctions.

Brotherton et al., 2022, Structure *30*, 697–706
May 5, 2022 © 2022 The Authors. Published by Elsevier Ltd.
https://doi.org/10.1016/j.str.2022.02.010

CellPress

Structure

CellPress
OPEN ACCESS

Article

Conformational changes and CO_2-induced channel gating in connexin26

Deborah H. Brotherton,[1] Christos G. Savva,[2] Timothy J. Ragan,[2] Nicholas Dale,[1,*] and Alexander D. Cameron[1,3,*]
[1]School of Life Sciences, University of Warwick, Gibbet Hill Road, CV4 7AL Coventry, UK
[2]Leicester Institute of Structural and Chemical Biology, Department of Molecular and Cell Biology, University of Leicester, Lancaster Road, LE1 7HB Leicester, UK
[3]Lead contact
*Correspondence: a.cameron@warwick.ac.uk (A.D.C.), n.e.dale@warwick.ac.uk (N.D.)
https://doi.org/10.1016/j.str.2022.02.010

SUMMARY

Connexins form large-pore channels that function either as dodecameric gap junctions or hexameric hemichannels to allow the regulated movement of small molecules and ions across cell membranes. Opening or closing of the channels is controlled by a variety of stimuli, and dysregulation leads to multiple diseases. An increase in the partial pressure of carbon dioxide (PCO_2) has been shown to cause connexin26 (Cx26) gap junctions to close. Here, we use cryoelectron microscopy (cryo-EM) to determine the structure of human Cx26 gap junctions under increasing levels of PCO_2. We show a correlation between the level of PCO_2 and the size of the aperture of the pore, governed by the N-terminal helices that line the pore. This indicates that CO_2 alone is sufficient to cause conformational changes in the protein. Analysis of the conformational states shows that movements at the N terminus are linked to both subunit rotation and flexing of the transmembrane helices.

INTRODUCTION

Twenty connexin genes have been identified in the human genome (Abascal and Zardoya, 2013). Connexins form hexameric plasma-membrane channels, or hemichannels, that can dock together to give dodecameric gap junctions. Gap junctions provide a direct aqueous passageway between cells, with functions independent of hemichannels (Pearson et al., 2005; Stout et al., 2004; Weissman et al., 2004). Connexin mutations underlie many different pathologies affecting all organ systems of the body (Garcia et al., 2016). Mutations of Cx26 constitute a leading cause of congenital deafness (Xu and Nicholson, 2013). Several rare, but dominant, mutations cause a severe syndromic disease: keratitis ichthyosis deafness syndrome (KIDS) (Xu and Nicholson, 2013).

The conductance of gap junctions and hemichannels can be modulated by voltage (Valiunas, 2002; Young and Peracchia, 2004), pH (Bevans and Harris, 1999; Khan et al., 2020; Yu et al., 2007), and, in some instances, by intracellular Ca^{2+} (Peracchia, 2004). We have discovered that a small group of closely related β-connexins can be directly modulated by modest physiologically relevant changes in the partial pressure of CO_2 (PCO_2) in arterial blood (Huckstepp et al., 2010a; Meigh et al., 2013). The effects of CO_2 on Cx26 gap junctions and hemichannels, respectively, are diametrically opposite: in hemichannels, CO_2 causes channel opening (de Wolf et al., 2017; Dospinescu et al., 2019; Huckstepp et al., 2010a; Meigh et al., 2013), whereas in gap junctions, CO_2 causes channel closure (Nijjar et al., 2021). Physiological and mutational analyses suggest that this CO_2 sensitivity is independent of pH (Huckstepp et al., 2010a; Meigh et al., 2013), is intrinsic to the protein (Meigh et al., 2013), and depends on a specific lysine (Dospinescu et al., 2019; Huckstepp et al., 2010a; Meigh et al., 2013, 2015). Binding of CO_2 to this specific lysine of Cx26 is physiologically important, as it contributes nearly half of the centrally generated chemosensory regulation of breathing to modest levels of hypercapnia (Huckstepp et al., 2010b; van de Wiel et al., 2020).

Several structures of Cx26 gap junctions have previously been reported, solved by both X-ray crystallography (Bennett et al., 2016; Maeda et al., 2009) and electron microscopy (Khan et al., 2020; Oshima et al., 2007) at low to modest resolution. The hemichannel is composed of 6 connexin subunits arranged around a central funnel. Each subunit consists of 4 transmembrane helices (TM1–4), an N-terminal helix, which lines the funnel entrance, a cytoplasmic loop, two extracellular loops, and a short C-terminal tail. While residues in the extracellular loops, which are involved in hemichannel docking, are generally well ordered among the solved structures, residues on the cytoplasmic side, including the N-terminal helix and the cytoplasmic loop, are much less well defined. Together with structures of other connexins (Flores et al., 2020; Lee et al., 2020; Myers et al., 2018), we are building up a picture of how these proteins are regulated. There remain, however, conflicting ideas of how they open and close in response to various signals. Original ideas, based on low-resolution electron crystallography, posited a mechanism involving subunit rotation (Unwin and Ennis, 1984). However,

This is an open access article under the CC BY license (http://creativecommons.org/licenses/by/4.0/).

the presence of a flexible N-terminal helix in the pore funnel has led to the idea that this may form a movable plug to block and open the channel (Maeda et al., 2009; Oshima et al., 2007, 2011; Yue et al., 2021). Yeager and co-workers suggested that regulation of Cx26 by extracellular Ca^{2+} was conferred by an electrostatic mechanism involving very minor modifications to the protein (Bennett et al., 2016), while in looking at regulation by pH, the same group have proposed a ball-and-chain mechanism involving dramatic conformational changes in which the N terminus completely refolds (Khan et al., 2020).

Here, we report high-resolution structures of Cx26, solved by cryoelectron microscopy (cryo-EM), from protein vitrified in buffers containing three levels of PCO_2 at constant pH. We show that CO_2 by itself is sufficient to change the conformation of the protein. Analyses of the conformational variability among these structures show that movements of the transmembrane helices, in particular, TM2, are correlated with the position of the N-terminal helix within the pore.

RESULTS

Cryo-EM structures of Cx26 under different levels of PCO_2

We first collected data from Cx26 vitrified in a CO_2/bicarbonate (HCO_3^-) buffer corresponding to a PCO_2 level of 55 mmHg and a pH of 7.4. This resulted in 3D reconstructions with nominal resolutions of 2.5 Å as defined by gold-standard Fourier shell correlations (FSCs) (Rosenthal and Henderson, 2003; Scheres, 2012) without applying symmetry and 2.2 Å when dihedral group 6 (D6) symmetry, consistent with the dodecameric structure of the gap junction, was used (Figure S1; Table S1). Remarkably, this resolution, limited by the pixel size during data collection, was much higher than similar preparations of protein vitrified in HEPES, where we could only obtain resolutions of approximately 4.5 Å, more similar to those observed by others (Khan et al., 2020). From this, we inferred that the CO_2/HCO_3^- buffer was stabilizing the protein. Encouraged by this, we therefore collected two further datasets at higher (90 mmHg) and lower (20 mmHg) PCO_2 levels. To ensure that differences between the two structures were confined as much as possible to only the PCO_2 levels, the protein was from the same preparation, being divided only in the final buffer-exchange step. The buffers were chosen to ensure that the pH and ionic strength did not differ (see STAR Methods and Figure S2). These datasets, collected with a smaller pixel size, gave reconstructions at even higher resolutions than those of the first data collected (1.9 Å for the 90 mmHg PCO_2 data and 2.1 Å for the 20 mmHg PCO_2 data with D6 symmetry; 2.1 Å and 2.7 Å, respectively, without symmetry; Figures S3 and S4; Table S1). While it was noted that there was some asymmetry in reconstructions, when symmetry was not applied, it was difficult to draw out any more detailed structural information than was seen in the D6 maps.

In all datasets, the overall quality of the density in the D6 reconstructions is very similar. On the extracellular side of the protein, the density is excellent, with clearly defined side chains, water molecules, and density consistent with the hydrocarbon chains of lipid or detergent (Figures 1 and S5–S7). On the other hand, the cytoplasmic side of the protein is less well defined, and there is no consistent density for the cytoplasmic loop between TM2 and TM3. In all maps, we observe density, which we have been unable to unambiguously assign, that protrudes from near the top of the visible density of TM3 toward the N-terminal helix (Figures S5–S7). In the structure of a related connexin, Cx46/50, more distinctive density in a similar position has been modeled with residues from the loop preceding TM3 (Flores et al., 2020). This corresponds to residues 127 to 130 of Cx26 and would thus be adjacent to the residues implicated in regulation by CO_2 (Dospinescu et al., 2019; Huckstepp et al., 2010a; Meigh et al., 2013, 2015). In structural predictions of Cx26 by both AlphaFold (Jumper et al., 2021) and RoseTTAFold (Baek et al., 2021), this region is modeled, albeit with a low confidence level, in a similar fashion, rather than by the continuation of the alpha helix that is present in the Cx26 crystal structure (Figure S8). It is possible that the density we observe also corresponds to these residues. We also see density reminiscent of a very short hydrocarbon chain of lipid or detergent lining the pore wall between the N terminus and TM1 and TM3 in all maps (Figures S5–S7). It is possible that the density is due to the dodecyl β-D-maltoside (DDM) that may have inserted into the pore during solubilization and became anchored through the interaction of its hydrocarbon chain with the hydrophobic wall of the funnel.

The major difference in the connectivity of the density associated with the different levels of PCO_2 concerns the density for the N-terminal helix (Figure 2). At the higher levels of PCO_2, the N-terminal helices fold into the pore such that there is a narrow constriction where all of the N-terminal helices from the six subunits meet (Figure 1). The density for these helices is more fragmented for the structure at low PCO_2, indicative of more flexibility of the helices associated with these particles (Figure 2).

Comparison with other Cx26 structures

After refinement of the structures against the respective D6 averaged maps associated with each level of CO_2, the coordinates look very similar, apart from at the N terminus. The resolution is much higher than has been seen for previous structures of Cx26 (Bennett et al., 2016; Khan et al., 2020; Maeda et al., 2009), enabling the protein to be modeled more accurately. Relative to these structures, the most interesting differences are observed at the N terminus and in TM1 (Figures 3 and S8). TM1 lines the inner surface of the cytoplasmic funnel (Figure 1). In other structures of Cx26, it has been built as a regular helix from residues Lys22 to Glu42 where it kinks as it turns into a 3_{10} helix (Bennett et al., 2016; Khan et al., 2020; Maeda et al., 2009). This region has been described as the TM1/extracellular loop 1 (TM1/E) interface. In our structures, the conformation of residues Val37 to Glu42 differs from that seen in previously published structures. Given that these residues are located within the pore, are adjacent to the constriction formed by the N termini (Figures 3 and S8), and are critical for function (Bennett et al., 2016; Verselis et al., 1994; Xu and Nicholson, 2013), we investigated whether this was a true conformational variation among the structures or whether it could have arisen from difficulties in map interpretation in the lower-resolution structures. We therefore reanalyzed the data from the previous crystal structures (see STAR Methods). While we could not unambiguously distinguish between these scenarios for the crystal structure from Maeda et al. (2009), the structures published by

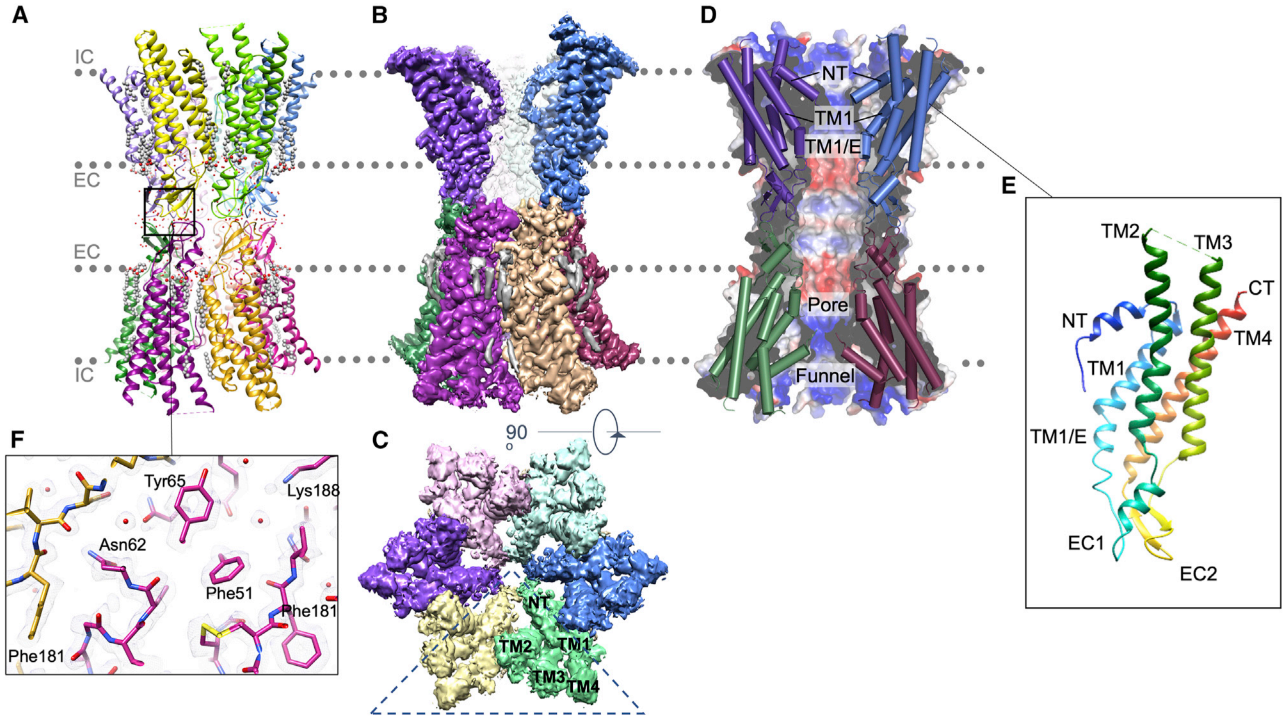

Figure 1. Structure of Cx26 solved by cryo-EM in CO_2/HCO_3^- buffer at 90 mmHg CO_2

(A) Ribbon diagram of connexin26, colored by subunit. Water molecules are indicated as red spheres, and lipid and detergent are shown as gray and red spheres. The membranes associated with the two opposed hemichannels are indicated by the dotted gray lines. IC, intracellular face; EC, extracellular face.
(B) Coulomb shell for the 90 mmHg CO_2 structure. The volume has been colored according to the associated subunit as shown in (A), with lipid and detergent chains in gray. Two subunits have been removed to expose the central channel and the N-terminal helices forming a funnel into the central opening.
(C) As (B) but viewed from the cytoplasmic side looking into the pore. The N-terminal helices fold into the pore to form a narrow restriction. The dashed triangle indicates the two subunits removed in view (B). Labels NT and TM1-4 indicate the positions of the structural elements within one subunit.
(D) View of the central channel through the gap junction indicating the electrostatic surface potential. Two subunits from each hemichannel are shown as cylinders. The N terminus is located at the mouth of the funnel, TM1 forms the wall of the funnel, and TM1/E bounds the pore of the channel.
(E) Ribbon diagram in the same orientation as the blue subunit in (D), colored from blue at the N terminus to red at the C terminus. Labels NT, EC1–2, and TM1–4 indicate the positions of the structural elements within one subunit.
(F) Density in the extracellular loops EC1 and EC2, which are involved in docking of the hemichannels. Carbon atoms are colored by chain, as shown in (A).

Bennett et al. (2016) were consistent with a conformational change (Figure S8).

The only other moderate resolution structure of Cx26 where there is distinct density for the N terminus is the crystal structure solved by Maeda et al. (2009). Comparing this structure with our structure at 90 mmHg PCO_2 shows clear differences in this region. Firstly, the trajectory taken by the N terminus into the pore differs slightly in the two structures, giving the constriction at the bottom a slightly different aperture (Figure 3). Secondly, the link between the N terminus and TM1 has been modeled farther from the funnel entrance in the crystal structure, incorporating the density that we observe to link TM3 and the N terminus (Figure 3). It has been shown that this conformation is not stable in molecular dynamics simulations (Myers et al., 2018), and, while unambiguous interpretation of the density in this area in the crystal structure is difficult, inspection of the respective maps shows that there are differences in how the N-terminal helix is situated in the pore with respect to the 90 mmHg PCO_2 Cx26 structure.

Structures have also been reported for two other connexins, the Cx46/50 (Flores et al., 2020; Myers et al., 2018) gap junction and the Cx31.3 (Lee et al., 2020) hemichannel. The core regions of these proteins, which correspond to the complete sequence of Cx26, have 50% and 35% sequence identity to human Cx26, respectively, and the profiles of the pores are very similar to Cx26, as seen in Figure 1A. However, whereas in Cx46/50, the N terminus (residues 1–16) is tucked back against the hydrophobic wall of the funnel, giving a much more open structure, in Cx31.3, the N-terminal helix is located closer to the cytoplasmic entrance such that it forms a lid with a narrow constriction of ~13 Å level with the cytoplasmic end of the transmembrane helices (Lee et al., 2020) rather than a plug within the funnel (Figure 3).

Conformational variability in Cx26 at fixed PCO_2

The cytoplasmic part of Cx26 is clearly flexible as judged, not only from the density and structural comparison but also from elastic network modeling, where considerable flexing of the subunits and transmembrane helices can be observed (Meigh et al., 2013). As cryo-EM datasets can harbor a range of conformations, we analyzed particle subsets in two ways. First, we used variability analysis as implemented in cryoSPARC (Punjani and Fleet, 2021; Punjani et al., 2017) (Videos S1, S2, and S3). A

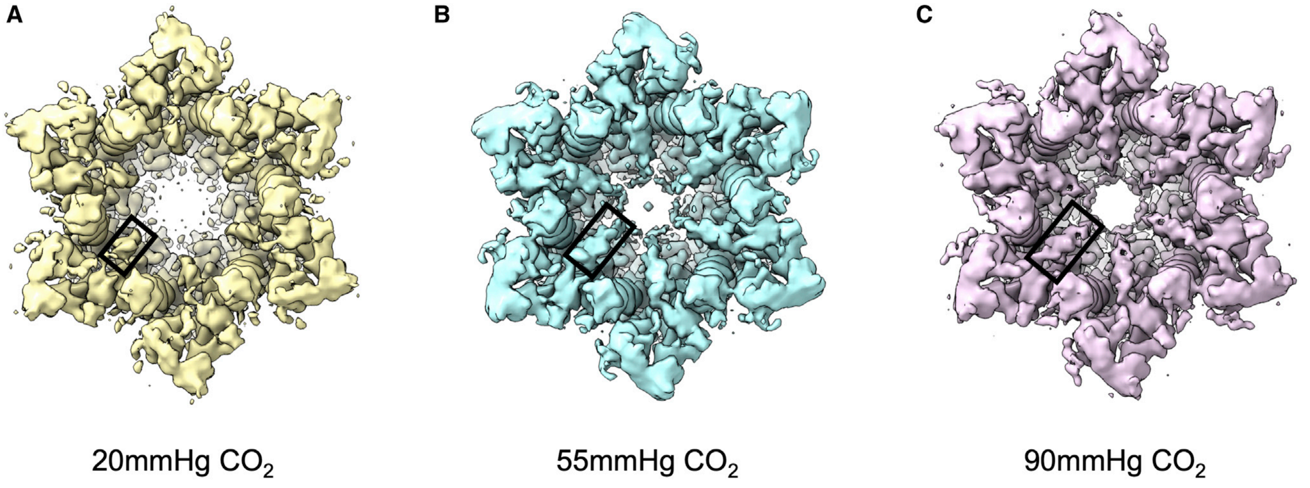

Figure 2. Variation in N-terminal density depending on levels of PCO_2

(A–C) View from the cytoplasmic side of the channel. At low PCO_2, the density for the N terminus is less defined, giving an apparently more open pore: (A) 20 mmHg (B), 55 mmHg (C), and 90 mmHg. Refinement and reconstructions were carried out using datasets of 125,000 particles with D6 symmetry. Maps have been low-pass filtered to 3 Å to minimize noise, and thresholds have been matched based on the density in the EC1/2 region. In each figure, one of the six copies of the N-terminal helices has been highlighted with a black rectangle.

variety of analyses were carried out selecting the hemichannel with the use of a soft-edged mask. Consistently, the most variation was seen at the N termini and in the cytoplasmic sides of the transmembrane helices. In the respective maps derived from data collected at 55 and 90 mmHg PCO_2, the N termini oscillated between a conformation where the helices met together in the core of the funnel and a position where the N termini were less defined in the density but pulled back from the pore (Videos S1 and S2). Together with this, the cytoplasmic portions of the transmembrane helices flexed with the greatest movement seen for TM2. In maps derived from data collected from protein vitrified at under 20 mmHg PCO_2 conditions, the general trend was the same, but the N termini were never as well defined as those corresponding to higher PCO_2, indicative of more mobility of the N termini (Video S3).

This analysis gave some idea of the variability within the datasets. We next sought to use a process of particle expansion, subtraction, and masked, fixed-angle classification in Relion (Scheres, 2012; Zivanov et al., 2018) to investigate whether we could select out particle sets with more consistent conformations in these regions (see STAR Methods). Firstly, we carried out an analysis with C6 symmetry, selecting out the cytoplasmic side of the protein with the use of a mask. The analysis for the data corresponding to the PCO_2 of 90 mmHg was the most interesting. Here, out of the eight given classes, the majority of particles divided into three classes, which, in order of particle number, result in maps with nominal resolutions of 2.0, 2.1, and 2.6 Å (Figures S9 and S10). Overall, the maps derived from particles belonging to the first and third classes looked similar in terms of the positions of the transmembrane helices and the aspect of the N-terminal helix (Figure S9). The second class, however, had a much better defined N terminus extending to a solid ring in the center of the funnel (Figures 4A and S9). It also had the most distinctively different conformation of the cytoplasmic tips of the transmembrane helices (Figure 4). A comparison of the maps derived from particles in classes 1 and 2, respectively, indicates there is a rotation of the whole cytoplasmic region of the protein relative to the extracellular side, with a flexing of TM2, in particular (Figures 4B and 4C; Video S4). This movement appears to be coupled to how well the N termini are defined in the density and has the aspect of an iris-like movement. The detergent-like density in the pore stays constant, and although the link between TM3 and the N-terminal helix also remains, its appearance changes (Figure 4; Video S4). The positions of the transmembrane helices in the D6 refined structure resembled most the top class from this classification. When we carried out a similar analysis with the lower particle number containing 20 and 55 mmHg PCO_2 datasets, we only obtained one class in each case with sufficient particles to give high-resolution maps. In each case, the positions of the transmembrane helices were very similar to the D6 refinements of the full datasets.

To understand the conformational variation within the 90 mmHg PCO_2 data in more detail, structures were built and refined from the maps associated with the top two classes (see Table S3). Comparing the two resulting structures (denoted hereafter as structure Class 1 [C1] and C2) shows that the cytoplasmic part of TM2 flexes at Pro 87 (Figure 4) such that there is an almost rigid body rotation of 14° of the C terminal part of the helix from residues Ala88 to Glu101. This region of TM2 is located between the loop linking the N-terminal helix with TM1 (residues 14 to 18) of the same respective subunit and the top of TM1 of the neighboring subunit. In structure C2, where the N terminus is more defined, TM2 is straighter with interactions between Val95 and His16, though the latter is not well defined in the density. In Structure C1, TM2 is bent toward TM1 of the neighboring subunit so that there is an interaction between Leu90 of TM2 and Trp24 of TM1. Morphing between these structures indicates that as TM2 moves, the N terminus and the following residues, including the first few residues of TM1, also change position (Video S5). The density associated with the side chains in this region is not well defined in either map.

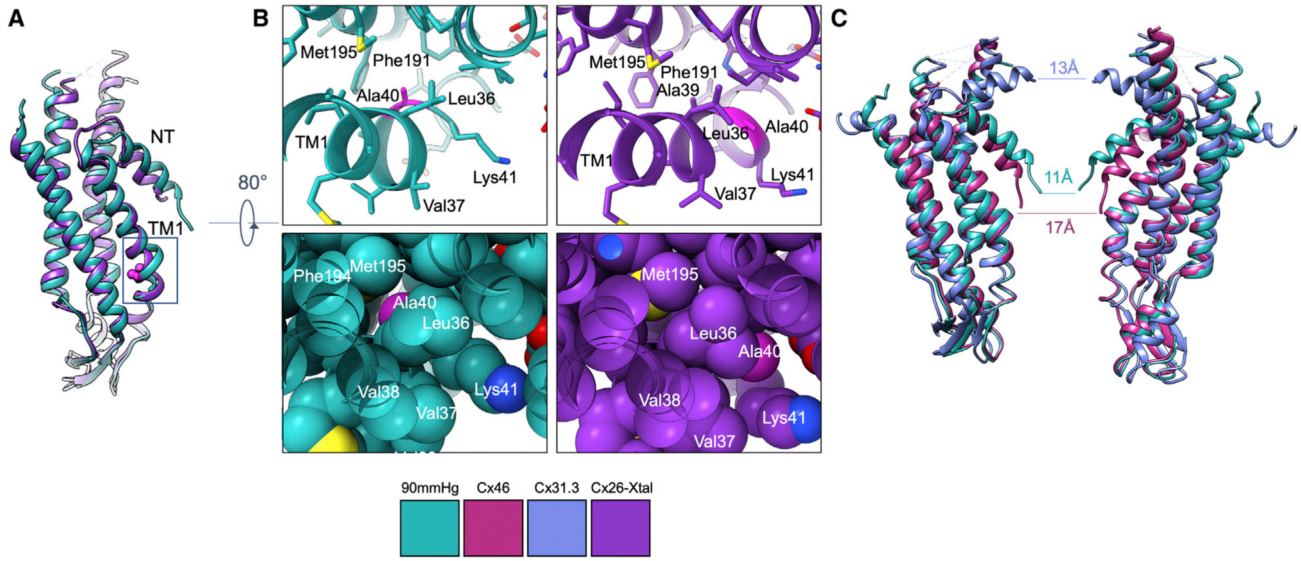

Figure 3. Comparison of the 90 mmHg PCO_2 Cx26 structure with other connexins

(A) Superposition of the 90 mmHg PCO_2 structure (sea green) with the Cx26 crystal structure (PDB: 2ZW3, purple) highlighting the differences in both the N terminus (NT) and the first transmembrane helix (TM1) between the two structures. The view highlights the differences in the connection of the N-terminal helix to TM1. For both structures, Ala40 is depicted in a ball-and-stick representation and colored magenta.

(B) Close up of the area around Ala40 boxed in (A), highlighting the change in position of Ala40. Left: the 90 mmHg PCO_2 Cx26 in stick (top) and sphere (bottom) representations. Right: the crystal structure (PDB: 2ZW3) in the same view.

(C) Comparison of the position of the N-terminal helices in the reported connexin structures. Two diametrically opposite subunits of a hemichannel are shown with the 90 mmHg PCO_2 Cx26 in sea green, Cx46 (PDB: 7JKC) in burgundy, and Cx31.3 (PDB: 6L3U) shown in sky blue. The distances represent the approximate diameter of the aperture formed in each case, based on the positions of the respective Cα atoms at the N terminus.

Overall, the conformational change has the aspect of a rotation of the cytoplasmic region of the protein with respect to the extracellular side.

Effect of PCO_2 on conformational distribution of Cx26

Clearly, the above classification procedure resulted in our being able to discriminate distinct conformations of the protein at a fixed level of PCO_2. However, given that the analysis of the 20 and 55 mmHg PCO_2 data was hampered by low particle numbers in all but the top classes, the question remained as to how differing levels of PCO_2 could alter the conformation of Cx26. Our inference from all the maps we examined, irrespective of particle number, lowpass resolution filter, masking, etc., was that the density associated with the N terminus was more distinct in the data obtained at high PCO_2 relative to that with low PCO_2. This suggests that elevated CO_2 might bias the conformations that are sampled by the N terminus. To examine this more quantitatively, we carried out a further classification without imposing symmetry and with the same numbers of particles from each PCO_2 dataset. As the N terminus is located at the interface of two subunits, we created a mask incorporating two subunits and again carried out a process of particle expansion, subtraction, and masked, fixed-angle classification as above, but using four classes (see STAR Methods). The distributions of particles in each of the classes can be seen in Figure 5. With the 90 mmHg PCO_2 dataset, the top class, including 78% of the particles, gave rise to a map where density for the N terminus could be seen to reach into the center of the pore, consistent with the D6 map. On the other hand, in the map associated with the top class from the 20 mmHg PCO_2 data, which contained 58% of the particles, the density associated with the N terminus was less defined and indicative of adopting a position nearer to the cytoplasmic side (Figure 5). Altogether, the results from this classification, as seen in Figure 5, support there being a difference between how well the N terminus is defined, with a trend from higher PCO_2 to lower PCO_2.

DISCUSSION

The structures of Cx26 reported here together with the analysis provide insight into the mechanism of these channels. Connexin gap junctions and hemichannels open and close in response to a variety of stimuli with models indicating either blockage through the N-terminal helices or a twisting iris-like movement (reviewed in Oshima, 2014). In our analysis, we see elements of both. Of the structures reported here, the narrowest constriction is observed for the 90 mmHg PCO_2 data where the diameter of the pore is approximately 11 Å, slightly narrower than that of the Cx26 crystal structure but much narrower than the pore seen in Cx46/50 (Figure 3). Only slight modifications of the side chains would be required to close the channel completely. The pore diameter is more similar to that of Cx31.3, where the N-terminal helices are closer to the cytoplasm (Figure 3). Whether the conformations seen in either Cx46/50 or Cx31.3 represent possible open and closed conformations that could be adopted in Cx26 is difficult to ascertain. The sequence identity, between the residues of the N-terminal helices in Cx31.3 and Cx26, is only 20%, with little conservation. In Cx46, where the N termini are more conserved

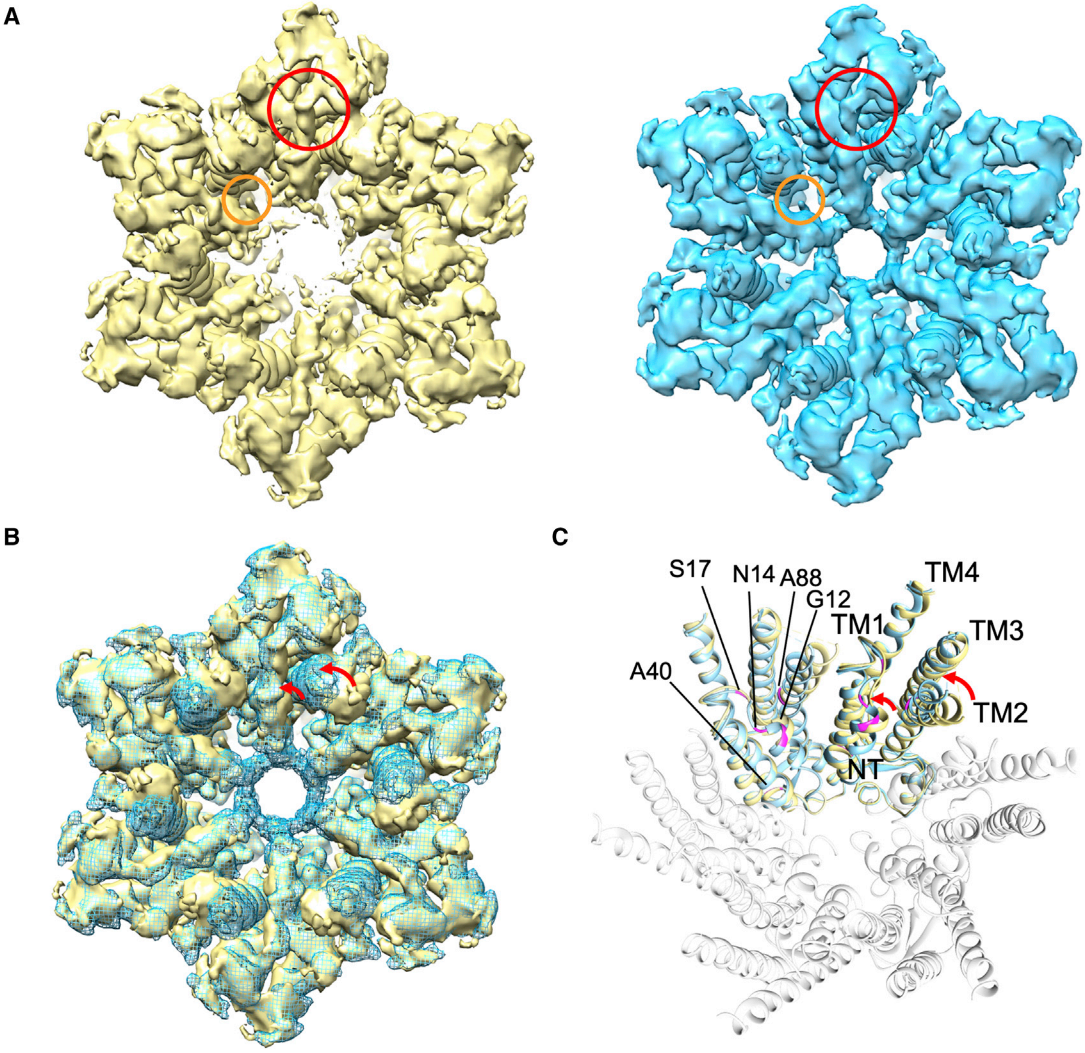

Figure 4. Comparison of the two most highly populated classes from the C6 classification of the 90 mmHg PCO_2 data

(A) Density associated with the first two classes: class 1 (yellow) and class 2 (cyan) (see Figure S9): The red circle shows the position of the extension from TM3, and the orange circle shows the position of the probable detergent in the pore.

(B) Superposition of the two maps from (A). The red arrows show the largest movements.

(C) Superposition of the two structures refined from the respective maps. The root-mean-square deviation (RMSD) between the two structures is 1.1 Å. Two of the six subunits are highlighted, colored as in (A) The KIDS mutations mentioned in the text are shown in magenta. See also Video S5.

with respect to Cx26 (30% sequence identity), Trp4 of the N-terminal helix slots between Ala39 of TM1 of one subunit and Ile34 and Leu35 of the next (Figure S11). In Cx26, the equivalent residue to Ala39 is replaced with the slightly bulkier residue valine (Val38). Leu35 is also replaced by a methionine (Met 34), for which mutation to alanine or threonine causes a loss of function (Oshima et al., 2003) and reduction in the response to CO_2 in hemichannels (de Wolf et al., 2016) and an apparent structural change in the N-terminal helices (Oshima et al., 2007). These conservative substitutions may affect the stability of the position of the N-terminal helix. This interpretation, however, does need to be taken with caution, as this is the site of the presumed detergent that we observe in the binding pocket (Figure S11). Were the detergent not present, we cannot rule out that the N termini would adopt a similar position to that seen in Cx46/50 in the open conformation rather than just becoming more flexible, as we observed at low PCO_2. More extensive lipid-like density in this region in Cx31.3 led to speculation that lipids may actually play a role in the mechanism (Lee et al., 2020) by analogy to a similar suggestion for innexins, gap-junction proteins in pre-chordates (Burendei et al., 2020).

The conformation that we observe at the TM1/E boundary in our structures, which includes Val38, discussed above, is more similar to the structures of both Cx46/50 (Flores et al., 2020) and Cx31.3 (Lee et al., 2020) than to the previously reported Cx26 crystal structures (Bennett et al., 2016; Maeda et al., 2009). From an examination of the density associated with each structure, this appears to be a distinct conformational difference rather than differences due to modeling. Why this should be is unclear. In our structures, there does appear to be some

CellPress
OPEN ACCESS

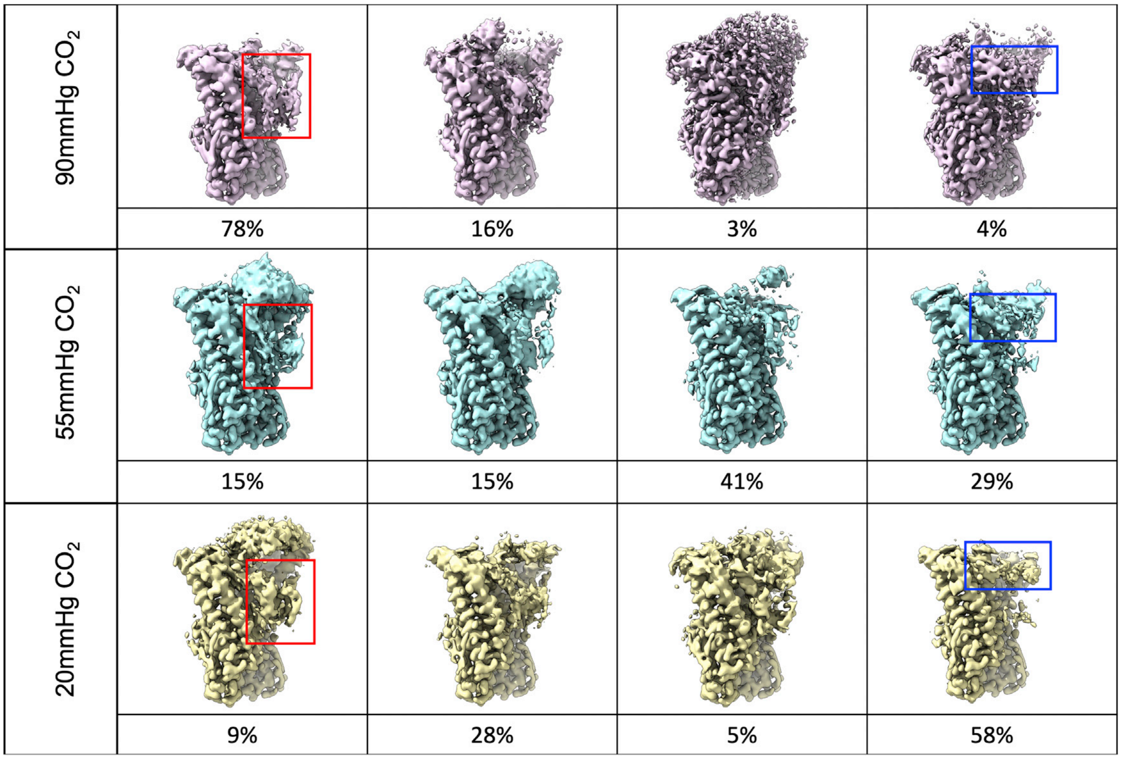

Figure 5. Classes observed with a 2-subunit mask at the three levels of PCO_2

From each dataset, 125,000 particles were randomly selected from the final D6 refinement. These were symmetry-expanded in D6, and the same mask was used for particle subtraction and 3D classification, with appropriate changes in pixel and box size for the 55 mmHg data. The percentage of particles in each class is shown below each class. Red boxes indicate the full, extended N-terminal helix (NT) position, and blue boxes indicate the truncated, raised NT location. The remaining two classes in each data set represent intermediates between these two extreme classes. The classes have been arranged in approximate order of the clarity of the density for the NT in the down position. This is clear for the two extremes but more ambiguous for the intermediate classes.

degree of flexibility in this area, and the variability analysis from cryoSPARC for the structure at low PCO_2 indicates a slight breathing at this point. This region includes the site of one of the KIDS mutations, Ala40 to valine (Xu and Nicholson, 2013). Hemichannels with this mutation are less responsive to extracellular Ca^{2+} (Sanchez et al., 2010), and both gap junctions and hemichannels are non-responsive to CO_2 (Cook et al., 2019; Nijjar et al., 2021). With the change in conformation relative to the previously published structures, Ala40 moves from a position that is more accessible to the pore to a more restricted position juxtaposed to TM2. This has important consequences for considering the effects of the mutation, as replacement of the alanine in this position with the bulkier valine is likely to perturb the structure and restrict any conformational changes necessary for activation, explaining why it may have more of an effect than might be expected from the previous structures (Figure 3). The region is also next to the Ca^{2+} binding site that is observed in the crystal structure with bound Ca^{2+}, with Glu42 acting as one of the Ca^{2+} ligands (Bennett et al., 2016). As no large-scale conformational differences were observed between Ca^{2+}-bound and free structures, an electrostatic mechanism was proposed to explain how Ca^{2+} was able to prevent the passage of ions through Cx26. Although this binding site for Ca^{2+} has been questioned (Lopez et al., 2016), it is also possible that the interaction of Glu42 with Ca^{2+} leads to the conformational changes we observe between the Ca^{2+}-bound structures and our structures (Figure S8). It can be speculated that any change in this area will affect the interaction with the N-terminal helix, as discussed above. It has previously been shown that Lys41, Glu42, and the N terminus all affect voltage gating (Verselis et al., 1994).

In addition to these conformational differences with respect to other published structures, all our analyses indicate a great deal of flexibility in the N terminus and in the cytoplasmic parts of the transmembrane helices. From the data associated with the 90 mmHg PCO_2, we teased out two distinct conformations of the protein. In one, the position of the N terminus is relatively well defined, such that it folds down to a narrow aperture within the funnel. In the other, the cytoplasmic region of the protein is rotated relative to the extracellular region, and the N terminus is less defined, giving the appearance of a more open pore. A comparison of the two structures also shows that TM2 twists around Pro87 and that the density between TM3 and the N terminus adopts a different trajectory. The rotation that is observed is, in fact, reminiscent of an early study on the regulation of Cx26 where pore closure was associated with a similar rotation of the cytoplasmic region (Unwin and Ennis, 1984). The presence of Pro87, which is highly conserved within connexins, has also been shown to be important for function (Suchyna et al., 1993), though whether the flexion of the helix that we observe stems from the proline itself is difficult to say given the quality of the

maps in this region. While we cannot say the range of movements seen represents the full ensemble required for occlusion or opening of the channel, owing to the lack of connectivity for the cytosolic loop and C terminus, there is clearly a series of positions that are intermediates, which together suggest how the protein may transition between open and closed states.

There are nine mutations that give rise to KIDS (Xu and Nicholson, 2013) (Figure 4). Ala40Val has been discussed above, and G45 is located nearby. Four of the mutations occur in the highly flexible regions of the protein near the flexion point of TM2 and in the linker between the N-terminal helix and TM1. These mutations involve substitution of bulkier side chains (Ala88Val and Gly12Arg, Asn14Lys/Tyr and Ser17Phe), which would likely impede the flexions required for channel gating (Video S5). Ala88 is situated next to Pro87 at the flexion point of TM2 and interacts with Val13 on the N-terminal helix between Gly12 and Asn14. Ala40Val, Ala88Val, and Asn14Lys have all been observed to abolish CO_2-mediated gap-junction closure (Nijjar et al., 2021).

We have previously reported that in a cellular environment, Cx26 gap junctions and hemichannels can be regulated by changes in PCO_2 (de Wolf et al., 2017; Dospinescu et al., 2019; Huckstepp et al., 2010a; Meigh et al., 2013). In this structural study, we clearly see an effect of the CO_2/HCO_3^- buffering system on the resolution of the maps that can be obtained relative to that observed when HEPES is used. This dramatic increase in resolution mirrors the improvement that was seen for Cx46/50 when cryo-EM was carried out with nanodiscs rather than amphipols (Flores et al., 2020; Myers et al., 2018) and suggests that the protein is being stabilized by CO_2/HCO_3^-. In cells, an increase in PCO_2 causes gap junctions to close (Nijjar et al., 2021). In our analysis, we observe that the most constricted pore is seen at the highest level of PCO_2, which is in broad agreement with the results in cells. It may be that the exact conformation of the N terminus is affected by the detergent in the pore; however, as this is present in all three datasets, we would not expect it to be the cause of the difference. Neither would we expect the difference to be caused by the particle numbers, as reducing the numbers of particles in the high PCO_2 datasets gives similar maps. Clearly, multiple conformations are being sampled in all three datasets collected, but it would appear that the more constricted pore is seen only in those associated with high PCO_2. The question must be asked why CO_2 would have this effect. A comprehensive series of mutational studies has led to the conclusion that the effect of CO_2 is mediated by the direct binding of CO_2 to Lys125 in a carbamylation reaction (Meigh et al., 2013, 2015; Nijjar et al., 2021; van de Wiel et al., 2020). Lys125 is located in the cytoplasmic loop, close to TM3. While we don't see Lys 125 in the density, we do see a link between TM3 and the N terminus, which, as described above, may reflect the position of Lys125. Any effect caused by the change in charge of Lys125 caused by carbamylation could therefore be transmitted to the N terminus through this link. Confirmation of this will require further structural studies. It is possible that we have not been able to fully carbamylate the lysine with the preparation of Cx26 in detergent and that we only increase the proportion of the protein carbamylated as we increase PCO_2. Regardless of this, we have shown how conformational changes in Cx26 can lead to channel opening and closing by movements of the N-terminal helix and that this effect is modified by buffers containing different levels of CO_2 at constant pH. This ties in with previous studies, which propose that the N terminus acts to gate the channel in response to signals such as voltage or pH (Maeda et al., 2009; Oshima et al., 2007, 2011; Yue et al., 2021) and that there is a link between the position of the N-terminal helix, TM2, and the cytoplasmic loop (Garcia et al., 2018; Valdez Capuccino et al., 2019). It is, however, very different from the "ball-and-chain mechanism" recently proposed, which is based on low-resolution structural information (Khan et al., 2020). This model relies on the N-terminal helix unwinding and plugging the central pore. To mediate the pH effect, profound acidification is required to cause the N-terminal helix to unfold and plug the channel, suggesting a function only in serious pathology. Here, we show that modest increases in PCO_2 are sufficient to cause conformational changes of the N-terminal helix plug in the absence of a dramatic change in pH.

STAR★METHODS

Detailed methods are provided in the online version of this paper and include the following:

- KEY RESOURCES TABLE
- RESOURCE AVAILABILITY
  - Lead contact
  - Materials availability
  - Data and code availability
- EXPERIMENTAL MODEL AND SUBJECT DETAILS
- METHOD DETAILS
  - Protein expression and purification
  - Cryo-EM sample preparation and data collection
  - Cryo-EM data processing
  - Variability analysis in cryoSPARC
  - Particle subtraction and masked classification in relion
  - Model building
  - Structural analysis
  - Revaluation of crystallographic data from previous publications
- QUANTIFICATION AND STATISTICAL ANALYSIS

SUPPLEMENTAL INFORMATION

Supplemental information can be found online at https://doi.org/10.1016/j.str.2022.02.010.

ACKNOWLEDGMENTS

We are grateful to Dr. Kyle Morris, Prof. Corinne Smith, and Dr. Saskia Bakker for discussion and advice and for the technical support from the School of Life Sciences, University of Warwick. We acknowledge the Midlands Regional Cryo-EM Facility, hosted at the Warwick Advanced Bioimaging Research Technology Platform, for use of the JEOL 2100Plus, and the Midlands Regional Cryo-EM Facility, hosted at Leicester Institute of Structural and Chemical Biology, for use of the FEI Titan Krios G3, both supported by MRC award reference MC_PC_17136. We thank the Leverhulme Trust (RPG-2015-090) and MRC (MR/P010393/1) for support.

AUTHOR CONTRIBUTIONS

The project was initiated and supervised by N.D. and A.D.C. Cloning, expression, purification, and grid preparation were carried out by D.H.B. Data collection was performed by C.G.S., T.J.R., and D.H.B. Data processing was done by D.H.B. and A.D.C. with guidance from T.J.R. and C.G.S. D.H.B. and A.D.C. refined the structures. A.D.C., D.H.B., and N.D. wrote the manuscript with contributions from all authors.

DECLARATION OF INTERESTS

The authors declare no competing interests.

Received: January 10, 2022
Revised: January 24, 2022
Accepted: February 14, 2022
Published: March 10, 2022

REFERENCES

Abascal, F., and Zardoya, R. (2013). Evolutionary analyses of gap junction protein families. Biochim. Biophys. Acta *1828*, 4–14.

Adams, P.D., Afonine, P.V., Bunkoczi, G., Chen, V.B., Davis, I.W., Echols, N., Headd, J.J., Hung, L.-W., Kapral, G.J., Grosse-Kunstleve, R.W., et al. (2010). PHENIX: a comprehensive Python-based system for macromolecular structure solution. Acta Crystallogr. D Biol. Crystallogr. *66*, 213–221.

Afonine, P.V., Grosse-Kunstleve, R.W., Echols, N., Headd, J.J., Moriarty, N.W., Mustyakimov, M., Terwilliger, T.C., Urzhumtsev, A., Zwart, P.H., and Adams, P.D. (2012). Towards automated crystallographic structure refinement with phenix.refine. Acta Crystallogr. D Biol. Crystallogr. *68*, 352–367.

Baek, M., DiMaio, F., Anishchenko, I., Dauparas, J., Ovchinnikov, S., Lee, G.R., Wang, J., Cong, Q., Kinch, L.N., Schaeffer, R.D., et al. (2021). Accurate prediction of protein structures and interactions using a three-track neural network. Science *373*, 871–876.

Bennett, B.C., Purdy, M.D., Baker, K.A., Acharya, C., McIntire, W.E., Stevens, R.C., Zhang, Q., Harris, A.L., Abagyan, R., and Yeager, M. (2016). An electrostatic mechanism for Ca(2+)-mediated regulation of gap junction channels. Nat. Commun. *7*, 8770.

Bevans, C.G., and Harris, A.L. (1999). Regulation of connexin channels by pH. Direct action of the protonated form of taurine and other aminosulfonates. J. Biol. Chem. *274*, 3711–3719.

Burendei, B., Shinozaki, R., Watanabe, M., Terada, T., Tani, K., Fujiyoshi, Y., and Oshima, A. (2020). Cryo-EM structures of undocked innexin-6 hemichannels in phospholipids. Sci. Adv. *6*, eaax3157.

Cook, J., de Wolf, E., and Dale, N. (2019). Cx26 keratitis ichthyosis deafness syndrome mutations trigger alternative splicing of Cx26 to prevent expression and cause toxicity in vitro. R. Soc. Open Sci. *6*, 191128.

de Wolf, E., Cook, J., and Dale, N. (2017). Evolutionary adaptation of the sensitivity of connexin26 hemichannels to CO_2. Proc. Biol. Sci. *284*, 20162723.

de Wolf, E., van de Wiel, J., Cook, J., and Dale, N. (2016). Altered CO_2 sensitivity of connexin26 mutant hemichannels in vitro. Physiol. Rep. *4*, e13038.

Delano, W.L. (2002). The PyMOL Molecular Graphics System (DeLano Scientific).

Dospinescu, V.-M., Nijjar, S., Spanos, F., Cook, J., de Wolf, E., Biscotti, M.A., Gerdol, M., and Dale, N. (2019). Structural determinants of CO_2-sensitivity in the β connexin family suggested by evolutionary analysis. Commun. Biol. *2*, 331.

Emsley, P., and Cowtan, K. (2004). Coot: model-building tools for molecular graphics. Acta Crystallogr. D Biol. Crystallogr. *60*, 2126–2132.

Flores, J.A., Haddad, B.G., Dolan, K.A., Myers, J.B., Yoshioka, C.C., Copperman, J., Zuckerman, D.M., and Reichow, S.L. (2020). Connexin-46/50 in a dynamic lipid environment resolved by CryoEM at 1.9 A. Nat. Commun. *11*, 4331.

Garcia, I.E., Prado, P., Pupo, A., Jara, O., Rojas-Gomez, D., Mujica, P., Flores-Munoz, C., Gonzalez-Casanova, J., Soto-Riveros, C., Pinto, B.I., et al. (2016). Connexinopathies: a structural and functional glimpse. BMC Cell Biol. *17*, 17.

Garcia, I.E., Villanelo, F., Contreras, G.F., Pupo, A., Pinto, B.I., Contreras, J.E., Perez-Acle, T., Alvarez, O., Latorre, R., Martinez, A.D., et al. (2018). The syndromic deafness mutation G12R impairs fast and slow gating in Cx26 hemichannels. J. Gen. Physiol. *150*, 697–711.

Goddard, T.D., Huang, C.C., and Ferrin, T.E. (2007). Visualizing density maps with UCSF Chimera. J. Struct. Biol. *157*, 281–287.

Goddard, T.D., Huang, C.C., Meng, E.C., Pettersen, E.F., Couch, G.S., Morris, J.H., and Ferrin, T.E. (2018). UCSF ChimeraX: meeting modern challenges in visualization and analysis. Protein Sci. *27*, 14–25.

Huckstepp, R.T., Eason, R., Sachdev, A., and Dale, N. (2010a). CO_2-dependent opening of connexin 26 and related beta connexins. J. Physiol. *588*, 3921–3931.

Huckstepp, R.T., id Bihi, R., Eason, R., Spyer, K.M., Dicke, N., Willecke, K., Marina, N., Gourine, A.V., and Dale, N. (2010b). Connexin hemichannel-mediated CO_2-dependent release of ATP in the medulla oblongata contributes to central respiratory chemosensitivity. J. Physiol. *588*, 3901–3920.

Jumper, J., Evans, R., Pritzel, A., Green, T., Figurnov, M., Ronneberger, O., Tunyasuvunakool, K., Bates, R., Zidek, A., Potapenko, A., et al. (2021). Highly accurate protein structure prediction with AlphaFold. Nature *596*, 583–589.

Khan, A.K., Jagielnicki, M., McIntire, W.E., Purdy, M.D., Dharmarajan, V., Griffin, P.R., and Yeager, M. (2020). A steric "Ball-and-Chain" mechanism for pH-mediated regulation of gap junction channels. Cell Rep. *31*, 107482.

Kucukelbir, A., Sigworth, F.J., and Tagare, H.D. (2014). Quantifying the local resolution of cryo-EM density maps. Nat. Methods *11*, 63–65.

Lee, H.J., Jeong, H., Hyun, J., Ryu, B., Park, K., Lim, H.H., Yoo, J., and Woo, J.S. (2020). Cryo-EM structure of human Cx31.3/GJC3 connexin hemichannel. Sci. Adv. *6*, eaba4996.

Lopez, W., Ramachandran, J., Alsamarah, A., Luo, Y., Harris, A.L., and Contreras, J.E. (2016). Mechanism of gating by calcium in connexin hemichannels. Proc. Natl. Acad. Sci. U S A. *113*, E7986–E7995.

Maeda, S., Nakagawa, S., Suga, M., Yamashita, E., Oshima, A., Fujiyoshi, Y., and Tsukihara, T. (2009). Structure of the connexin 26 gap junction channel at 3.5 A resolution. Nature *458*, 597–602.

Meigh, L., Cook, D., Zhang, J., and Dale, N. (2015). Rational design of new NO and redox sensitivity into connexin26 hemichannels. Open Biol. *5*, 140208.

Meigh, L., Greenhalgh, S.A., Rodgers, T.L., Cann, M.J., Roper, D.I., and Dale, N. (2013). CO(2)directly modulates connexin 26 by formation of carbamate bridges between subunits. Elife *2*, e01213.

Murshudov, G.N., Skubak, P., Lebedev, A.A., Pannu, N.S., Steiner, R.A., Nicholls, R.A., Winn, M.D., Long, F., and Vagin, A.A. (2011). REFMAC5 for the refinement of macromolecular crystal structures. Acta Crystallogr. D Biol. Crystallogr. *67*, 355–367.

Myers, J.B., Haddad, B.G., O'Neill, S.E., Chorev, D.S., Yoshioka, C.C., Robinson, C.V., Zuckerman, D.M., and Reichow, S.L. (2018). Structure of native lens connexin 46/50 intercellular channels by cryo-EM. Nature *564*, 372–377.

Nijjar, S., Maddison, D., Meigh, L., de Wolf, E., Rodgers, T., Cann, M.J., and Dale, N. (2021). Opposing modulation of Cx26 gap junctions and hemichannels by CO_2. J. Physiol. *599*, 103–118.

Oshima, A. (2014). Structure and closure of connexin gap junction channels. FEBS Lett. *588*, 1230–1237.

Oshima, A., Doi, T., Mitsuoka, K., Maeda, S., and Fujiyoshi, Y. (2003). Roles of Met-34, Cys-64, and Arg-75 in the assembly of human connexin 26. Implication for key amino acid residues for channel formation and function. J. Biol. Chem. *278*, 1807–1816.

Oshima, A., Tani, K., Hiroaki, Y., Fujiyoshi, Y., and Sosinsky, G.E. (2007). Three-dimensional structure of a human connexin26 gap junction channel reveals a plug in the vestibule. Proc. Natl. Acad. Sci. U S A. *104*, 10034–10039.

Oshima, A., Tani, K., Toloue, M.M., Hiroaki, Y., Smock, A., Inukai, S., Cone, A., Nicholson, B.J., Sosinsky, G.E., and Fujiyoshi, Y. (2011). Asymmetric configurations and N-terminal rearrangements in connexin26 gap junction channels. J. Mol. Biol. *405*, 724–735.

Pearson, R.A., Dale, N., Llaudet, E., and Mobbs, P. (2005). ATP released via gap junction hemichannels from the pigment epithelium regulates neural retinal progenitor proliferation. Neuron *46*, 731–744.

Peracchia, C. (2004). Chemical gating of gap junction channels; roles of calcium, pH and calmodulin. Biochim. Biophys. Acta *1662*, 61–80.

Pettersen, E.F., Goddard, T.D., Huang, C.C., Couch, G.S., Greenblatt, D.M., Meng, E.C., and Ferrin, T.E. (2004). UCSF Chimera–a visualization system for exploratory research and analysis. J. Comput. Chem. *25*, 1605–1612.

Punjani, A., and Fleet, D.J. (2021). 3D variability analysis: resolving continuous flexibility and discrete heterogeneity from single particle cryo-EM. J. Struct. Biol. *213*, 107702.

Punjani, A., Rubinstein, J.L., Fleet, D.J., and Brubaker, M.A. (2017). cryoSPARC: algorithms for rapid unsupervised cryo-EM structure determination. Nat. Methods *14*, 290–296.

Rohou, A., and Grigorieff, N. (2015). CTFFIND4: fast and accurate defocus estimation from electron micrographs. J. Struct. Biol. *192*, 216–221.

Rosenthal, P.B., and Henderson, R. (2003). Optimal determination of particle orientation, absolute hand, and contrast loss in single-particle electron cryomicroscopy. J. Mol. Biol. *333*, 721–745.

Sanchez, H.A., Mese, G., Srinivas, M., White, T.W., and Verselis, V.K. (2010). Differentially altered Ca2+ regulation and Ca2+ permeability in Cx26 hemichannels formed by the A40V and G45E mutations that cause keratitis ichthyosis deafness syndrome. J. Gen. Physiol. *136*, 47–62.

Scheres, S.H. (2012). RELION: implementation of a Bayesian approach to cryo-EM structure determination. J. Struct. Biol. *180*, 519–530.

Stout, C., Goodenough, D.A., and Paul, D.L. (2004). Connexins: functions without junctions. Curr. Opin. Cell Biol. *16*, 507–512.

Suchyna, T.M., Xu, L.X., Gao, F., Fourtner, C.R., and Nicholson, B.J. (1993). Identification of a proline residue as a transduction element involved in voltage gating of gap junctions. Nature *365*, 847–849.

Unwin, P.N., and Ennis, P.D. (1984). Two configurations of a channel-forming membrane protein. Nature *307*, 609–613.

Valdez Capuccino, J.M., Chatterjee, P., Garcia, I.E., Botello-Smith, W.M., Zhang, H., Harris, A.L., Luo, Y., and Contreras, J.E. (2019). The connexin26 human mutation N14K disrupts cytosolic intersubunit interactions and promotes channel opening. J. Gen. Physiol. *151*, 328–341.

Valiunas, V. (2002). Biophysical properties of connexin-45 gap junction hemichannels studied in vertebrate cells. J. Gen. Physiol. *119*, 147–164.

van de Wiel, J., Meigh, L., Bhandare, A., Cook, J., Nijjar, S., Huckstepp, R., and Dale, N. (2020). Connexin26 mediates CO_2-dependent regulation of breathing via glial cells of the medulla oblongata. Commun. Biol. *3*, 521.

Verselis, V.K., Ginter, C.S., and Bargiello, T.A. (1994). Opposite voltage gating polarities of two closely related connexins. Nature *368*, 348–351.

Weissman, T.A., Riquelme, P.A., Ivic, L., Flint, A.C., and Kriegstein, A.R. (2004). Calcium waves propagate through radial glial cells and modulate proliferation in the developing neocortex. Neuron *43*, 647–661.

Xu, J., and Nicholson, B.J. (2013). The role of connexins in ear and skin physiology - functional insights from disease-associated mutations. Biochim. Biophys. Acta *1828*, 167–178.

Young, K.C., and Peracchia, C. (2004). Opposite Cx32 and Cx26 voltage-gating response to CO2 reflects opposite voltage-gating polarity. J. Membr. Biol. *202*, 161–170.

Yu, J., Bippes, C.A., Hand, G.M., Muller, D.J., and Sosinsky, G.E. (2007). Aminosulfonate modulated pH-induced conformational changes in connexin26 hemichannels. J. Biol. Chem. *282*, 8895–8904.

Yue, B., Haddad, B.G., Khan, U., Chen, H., Atalla, M., Zhang, Z., Zuckerman, D.M., Reichow, S.L., and Bai, D. (2021). Connexin 46 and connexin 50 gap junction channel properties are shaped by structural and dynamic features of their N-terminal domains. J. Physiol. *599*, 3313–3335.

Zheng, S.Q., Palovcak, E., Armache, J.P., Verba, K.A., Cheng, Y., and Agard, D.A. (2017). MotionCor2: anisotropic correction of beam-induced motion for improved cryo-electron microscopy. Nat. Methods *14*, 331–332.

Zivanov, J., Nakane, T., Forsberg, B.O., Kimanius, D., Hagen, W.J., Lindahl, E., and Scheres, S.H. (2018). New tools for automated high-resolution cryo-EM structure determination in RELION-3. Elife *7*, e42166.

CellPress
OPEN ACCESS

STAR★METHODS

KEY RESOURCES TABLE

REAGENT or RESOURCE	SOURCE	IDENTIFIER
Bacterial and virus strains		
Sf9 Cells	Fisher Scientific	CAT# 10500343
Chemicals, peptides, and recombinant proteins		
Insect Xpress	Scientific laboratory Supplies/Lonza	CAT# LZBELN12-730Q
Histidine	Merck/Sigma	CAT# H6034-100G
n-Dodecyl β-maltoside (DDM-C)	Glycon Biochemicals GMBH	CAT# D97002-C-50g
cOmplete™, EDTA-free Protease Inhibitor Cocktail	Merck/Roche	CAT# 4693132001
AEBSF hydrochloride	Fisher Scientific/Acros	CAT# 10742885
Dithiothreitol	Fisher Scientific	CAT# 10592945
DNAse I	Merck/Roche	CAT# 10104159001
Econocolumn	Bio-Rad	CAT# 7372532
HisPur Ni-NTA Resin	Thermo Scientific/ Pierce	CAT# 88221
5/150 Superose 6 column	GE Healthcare Lifescience	CAT# 15383224
Quantifoil 0.6/1 300 mesh Au holey carbon	Quantifoil Micro Tools GMBH	CAT# N1-C11nAu30-50
UltrAuFoil 01.2/1.3 300 mesh Holey gold	Quantifoil Micro Tools GMBH	CAT# N1-A14nAu30-50
UltrAuFoil 0.6/1 300 mesh Holey gold	Quantifoil Micro Tools GMBH	CAT# N1-A11nAu30-50
Formvar/Carbon on copper 300mesh	EM resolutions Ltd.	CAT# FC300Cu100
Vivaspin-20 MWCO 100,000	Sartorius	CAT# VS2042
Vivaspin-500 MWCO 100,000	Sartorius	CAT# VS0141
Vivaspin-6 MWCO 100,000	Sartorius	CAT# VS0641
Slide-a-lyzer, 10 k cutoff	Fisher Scientific/ Pierce	CAT# 11809420
Custom mix 2.5 % CO_2/97.5 % N_2	BOC	CAT# 226782-V
Custom mix 10 % CO_2/90 % N_2	BOC	CAT# 226699-V
Custom mix 15 % CO_2/85 % N_2	BOC	CAT# 120163-V
Deposited data		
20 mmHg PCO2 dodecamer Cryo-EM Volume	This Paper	EMDB: EMD-13940
20 mmHg PCO2 dodecamer Atomic Coordinates	This Paper	PDB: 7QET
55 mmHg PCO2 dodecamer Cryo-EM Volume	This Paper	EMDB: EMD-13938
55 mmHg PCO2 dodecamer Atomic Coordinates	This Paper	PDB: 7QER
90 mmHg PCO2 Cryo-EM Volume	This Paper	EMDB: EMD-13937
90 mmHg PCO2 Atomic Coordinates	This Paper	PDB: 7QEQ
Class 1 Hexamer, 90 mmHg Cryo-EM Volume	This Paper	EMDB: EMDB-13944
Class 1 Hexamer, 90 mmHg Atomic Coordinates	This Paper	PDB: 7QEY
Class 2 Hexamer, 90 mmHg PCO2 Cryo-EM Volume	This Paper	EMDB: EMDB-13943
Class 2 Hexamer, 90 mmHg PCO2 Atomic Coordinates	This Paper	PDB: 7QEW
Cx26 crystal structure	Maeda et al. (2009)	PDB: 2ZW3
Cx26 with $Ca2^{+}$ crystal structure	Bennett et al. (2016)	PDB: 5ER7

(Continued on next page)

Continued		
REAGENT or RESOURCE	SOURCE	IDENTIFIER
Cx26 without Ca2$^+$ crystal structure	Bennett et al. (2016)	PDB: 5ERA
Sheep Cx46 cryo-EM structure	Flores et al. (2020)	PDB: 7JKC
Human Cx31.3 cryo-EM structure	Lee et al. (2020)	PDB: 6L3U
Recombinant DNA		
pFastbac-human connexin 26	Gift from Prof Tomitake Tsukihara and Prof Atsunori	N/A
Software and algorithms		
Relion 3.1	(Zivanov et al., 2018)	https://www3.mrc-lmb.cam.ac.uk/relion/index.php/Main_Page
MotionCorr2	(Zheng et al., 2017)	https://emcore.ucsf.edu/ucsf-software
Ctffind 4	(Rohou and Grigorieff, 2015)	https://grigoriefflab.umassmed.edu/ctffind4
Coot	(Emsley and Cowtan, 2004)	https://www2.mrc-lmb.cam.ac.uk/personal/pemsley/coot/
Phenix	(Adams et al., 2010)	https://phenix-online.org
CryoSPARC	(Punjani et al., 2017)	https://cryosparc.com
Resmap	(Kucukelbir et al., 2014)	http://resmap.sourceforge.net
Chimera	(Goddard et al., 2007)	https://www.cgl.ucsf.edu/chimera/
ChimeraX	(Goddard et al., 2018)	https://www.cgl.ucsf.edu/chimerax/

RESOURCE AVAILABILITY

Lead contact

Further information and requests for resources and reagents should be directed to and will be fulfilled by the lead contact, Alexander Cameron (a.cameron@warwick.ac.uk).

Materials availability

This study did not generate unique reagents.

Data and code availability

Cryo-EM density maps have been deposited in the Electron Microscopy DataBank (EMDB) and atomic coordinates have been deposited in the Protein Data Bank. They are publicly available as of the date of publication. Accession numbers are listed in the key resources table. This paper does not report original code. Any additional information required to reanalyze the data reported in this paper is available from the lead contact upon request.

EXPERIMENTAL MODEL AND SUBJECT DETAILS

Human connexin 26 protein (hCx26) was expressed by infecting *Spodoptera frugiperda* (Sf9) cells for 72 hours with a baculovirus carrying the hCx26 gene.

METHOD DETAILS

Protein expression and purification

Human connexin 26 protein (hCx26) was expressed with a thrombin-cleavable His(6) affinity tag on the C terminus using baculovirus in *Spodoptera frugiperda* (Sf9) cells. This construct was a gift from Prof Tomitake Tsukihara. Cells were harvested 72 hours post infection by centrifugation at 2500 x g in a Beckmann JLA 8.1000 rotor, cell pellets were snap frozen in liquid nitrogen, and stored at −80°C until purification. Cells were thawed in hypotonic lysis buffer, (10 mM sodium phosphate, 10 mM NaCl, 5 mM $MgCl_2$, 1 mM DTT, pH 8.0) DNAse I, cOmplete™ EDTA-free Protease Inhibitor Cocktail (Roche) and AEBSF were added according to manufacturer's instructions before use. After 30 minutes stirring at 4°C, the cells were broken using 40 strokes with a dounce homogeniser, and the membranes separated by ultracentrifugation for 1 hour at 4°C, 158000 x g. Membranes were resuspended in membrane resuspension buffer (25 mM sodium phosphate, 150 mM NaCl, 5 % glycerol, 1 mM DTT, pH 8.0). DNAse I, cOmplete™ EDTA-free Protease Inhibitor Cocktail and AEBSF were added according to manufacturer's instructions before use. This membrane suspension was diluted (to 400 ml) with solubilisation buffer (10 mM sodium phosphate, 300 mM NaCl, 5 % glycerol, 1 mM DTT, 1%

DDM (Glycon Biochemicals GmbH), pH 8.0) and incubated at 4°C for 3 hours, before a further 1 hour ultracentrifugation at 4°C, 158000 x g to remove insoluble material. The soluble material was batch bound to pre-equilibrated HisPur Ni-NTA resin (Thermo Scientific) overnight, and then poured into an econocolumn for subsequent manual washing and elution steps. Resin was washed with 5x CV wash buffer (10 mM sodium phosphate, 500 mM NaCl, 10 mM histidine, 5 % glycerol, 1 mM DTT, 0.1 % DDM, pH 8.0) before eluting hCx26 with elution buffer (10 mM sodium phosphate, 500 mM NaCl, 200 mM histidine, 5 % glycerol, 1 mM DTT, 0.1 % DDM, pH 8.0). Fractions containing hCx26 were dialysed overnight 4°C against (10 mM sodium phosphate, 500 mM NaCl, 5 % glycerol, 1 mM DTT, 0.03 % DDM, pH 8.0). For the protein used for electron microscopy thrombin (Sigma) 1:1 w/w was added during dialysis to remove the affinity label). hCx26 was then 0.2 μm filtered, concentrated in a vivaspin 100,000 MWCO and loaded onto a Superose 6 Increase 5/150 size exclusion chromatography column (GE Healthcare Lifescience) to remove thrombin and exchange the buffer to either 20 mm, 55 mm or 90 mmHg αCSF buffer (Huckstepp et al., 2010a) (*20 mmHg αCSF buffer*: 140 mM NaCl, 5 % glycerol, 1 mM DTT, 0.03 % DDM, 10 mM $NaHCO_3$, 1.25 mM NaH_2PO_4, 3 mM KCl, 1 mM $MgSO_4$, 2 mM $MgCl_2$; *55 mmHg αCSF buffer*: 100 mM NaCl, 5 % glycerol, 1 mM DTT, 0.03 % DDM, 50 mM $NaHCO_3$, 1.25 mM NaH_2PO_4, 3 mM KCl, 1 mM $MgSO_4$, 2 mM $MgCl_2$; *90 mmHg αCSF buffer*: 70 mM NaCl, 5 % glycerol, 1 mM DTT, 0.03 % DDM, 80 mM $NaHCO_3$, 1.25 mM NaH_2PO_4, 3 mM KCl, 1 mM $MgSO_4$, 4 mM $MgCl_2$).

Cryo-EM sample preparation and data collection

The hCx26 peak was concentrated to 3.5 mg/ml before being gassed with the correct amount of CO_2 to give a final pH of ~7.4 as described below for each buffer. 2.5%, 10% and 15% CO_2 in N_2 (BOC) were used for the 20, 55 and 90 mmHg buffers respectively. To ensure that the pH was consistent when the protein was vitrified in the relevant buffer, the following controls were applied.

Firstly, 10 ml of buffer was equilibrated with the correct percentage of CO_2 for the buffer and the pH checked using a pH meter. Phenol red solution (1:200) was then added to the samples of gassed and ungassed buffers to provide a reference colour for the pH. The volume of the protein to be used for vitrification was noted, and an equal volume of phenol red/buffer was added to two separate Eppendorf tubes, one of which was gassed until the colour matched an equal volume of the pre-gassed 10 ml sample. The time of gassing was noted, and the protein was treated identically (Figure S2). For both the 20 mmHg and 90 mmHg conditions the sample was left overnight dialysing against 60 ml buffer that was gassed with the correct concentration of CO_2 to give a pH of 7.4. Quantifoil 300 mesh gold grids (either 0.6/1 carbon film, 0.6/1 or 1.2/1.3 UltrAUfoil, Quantifoil Micro Tools GMBH) were glow discharged for 30 seconds prior to use. Vitrification of the protein in liquid ethane/propane at −180°C was carried out with a Leica GP2 automated plunge freezer with 3 μl protein per grid at 4°C, 95 % humidity, 6 seconds blotting in a CO_2/N_2 atmosphere appropriate for the buffer used. Grids were screened using a Jeol 2100plus microscope, and data were collected on an FEI Titan Krios G3 equipped with a K3 detector and BioQuantum energy filter using a 20 eV slit width. A dose rate of ~10 e/pix/sec on the detector was chosen with a final dose of between 40-45 e/Å^2 on the specimen. Data collections were prepared and run automatically using EPU2.X and aberration-free image shift (AFIS).

Cryo-EM data processing

Data were processed using Relion3.1-beta (Zivanov et al., 2018), using essentially the same protocol for the three data sets, accounting for the different pixel size. Micrographs were motion corrected using the version of MotionCor2 (Zheng et al., 2017) implemented in Relion, and CTFs were estimated using ctffind4 (Rohou and Grigorieff, 2015). Particles were picked using the Laplacian of Gaussian (LoG) picker, and poor, damaged, or junk particles were removed by serial rounds of 2D classifications with particles down-sampled to 4 Å/pixel. 3D classification was carried out in C1 with an initial model generated from a previous Cx26 cryo-EM reconstruction (unpublished data) with a low-pass filter of 30 Å. Multiple rounds of 3D classification in C1 resulted in 4 very similar classes. Exhaustive rounds of refinement, CTF refinement and polishing in Relion with unbinned particles were used to improve the resolution of the Coulomb shells until no further improvement was gained. Trials were made treating the classes individually or pooling them in C1, C2, C3, C6 and D6 symmetry. While variations were noted in the associated maps, it was not possible to identify different structural features. The resolution was estimated based on the gold standard Fourier Shell Coefficient (FSC) criterion (Rosenthal and Henderson, 2003; Scheres, 2012) with a soft solvent mask. Local Resolution estimation was carried out in ResMap (Kucukelbir et al., 2014).

Variability analysis in cryoSPARC

The refined particles associated with each data set were downsampled to 1.586 Å/pixel and imported into cryoSPARC (Punjani et al., 2017). An *ab-initio* reconstruction was made in C1 and the particles refined in cryoSPARC using D6 symmetry to give maps with an estimated resolution of 3.24 Å. Particle expansion was carried out with D6 symmetry. These were then subjected to variability analysis in cryoSPARC with a filter resolution of 4.5 Å and a mask over the six subunits of one of the two docked hemichannels that form the gap junction. The results were displayed as a simple movie of 20 frames shown in Chimera (Goddard et al., 2007; Pettersen et al., 2004). Analysis was also carried out with masks covering single or neighbouring subunits, but these did not appear to give any advantage over the hemichannel mask.

Particle subtraction and masked classification in relion

Hemichannel classification with imposed C6 symmetry

A mask was created in Chimera (Goddard et al., 2007; Pettersen et al., 2004) based on the best-defined region of the D6-refined dodecameric model, and a soft edge added in relion_mask_create. D6-refined particle sets for each dataset were further refined with this mask. These particles were then symmetry expanded in D1. A mask was created in Chimera (Goddard et al., 2007; Pettersen et al., 2004) based on the least-defined region of the D6-refined dodecameric model, and a soft edge added in relion_mask_create. This mask was then used in particle subtraction and 3D classification without image alignment. The top 3 classes from each dataset were selected, and the original particles recovered. These were refined with C6 symmetry imposed, using a hemichannel mask and limited initial angular sampling. The resolution of the maps was estimated by gold standard Fourier Shell Correlations.

Two-subunit classification without imposed symmetry

For consistency among the three data sets, which contain different numbers of particles, 125,000 particles were randomly selected from each of the 55 mmHg and 90 mmHg PCO_2 D6 refined particle sets to match the particle numbers in the 20 mmHg PCO_2 data set. These were then symmetry expanded in D6. A mask was created in Chimera (Goddard et al., 2007; Pettersen et al., 2004) based on two neighbouring subunits of the refined model and a soft edge added in relion_mask_create. This was then used in particle subtraction and 3D classification without image alignment. Reconstructions were created with half subsets of each class and the resolution was estimate with relion_postprocess.

Model building

Initial model building was carried out in Coot (Emsley and Cowtan, 2004) with the D6 reconstructions of the 55 mmHg CO_2 data with an initial model based on the crystal structure of Cx26 (Maeda et al., 2009). Residues at the C-terminus and the cytoplasmic loop between 102 and 128 could not be observed and were not included in the model. While the density clearly showed the position of the N-terminal helix the side chains were not well defined and modelling of the first residues is ambiguous. For this reason, we have omitted the first three residues in the final PDB file. Real space refinement in Phenix (Adams et al., 2010) was carried out with NCS constraints. Water molecules were also added during refinement with further curation of the water molecules added in Coot. Lipids or detergents were clearly visible in the density, though the exact nature of the head groups is ambiguous. The head-groups have been omitted in the final structure. Refinement of the 20 mmHg and 90 mmHg structures was carried out starting from a partially refined structure of the 55 mmHg CO_2 structure using a similar protocol. For the structure derived from the second class of the C6 classification of the 90 mmHg PCO_2 structure residues were tentatively modelled for the N-terminal helix starting from Asp2.

Structural analysis

All structural images shown in this paper were generated in Chimera (Goddard et al., 2007; Pettersen et al., 2004) or Chimera X (Goddard et al., 2018) except for Figure 1D, which was created in PyMol (Delano, 2002). Superpositions were carried out in Chimera such that only matching C_α pairs within 2 Å after superposition were included in the matrix calculation.

Revaluation of crystallographic data from previous publications

The structure derived from the 90 mmHg data set was refined against the data sets deriving associated with the structures from Maeda et al. (PDB:2ZW3) (Maeda et al., 2009) and Bennet et al. (Bennett et al., 2016) (PDB: 5er7, PDB 5era) using both Refmac5 (Murshudov et al., 2011) and Phenix.refine (Afonine et al., 2012). The resulting maps were examined in Coot (Emsley and Cowtan, 2004).

QUANTIFICATION AND STATISTICAL ANALYSIS

Cryo-EM reconstructions were performed as described in the method details using cryoSPARC (v2.14.2) and Relion 3.1. Phenix (1.18.2) was used for refinement. Statistics of data collection and refinement are given in Tables S1–S3.

Structure, Volume *30*

Supplemental Information

Conformational changes and CO_2-induced channel gating in connexin26

Deborah H. Brotherton, Christos G. Savva, Timothy J. Ragan, Nicholas Dale, and Alexander D. Cameron

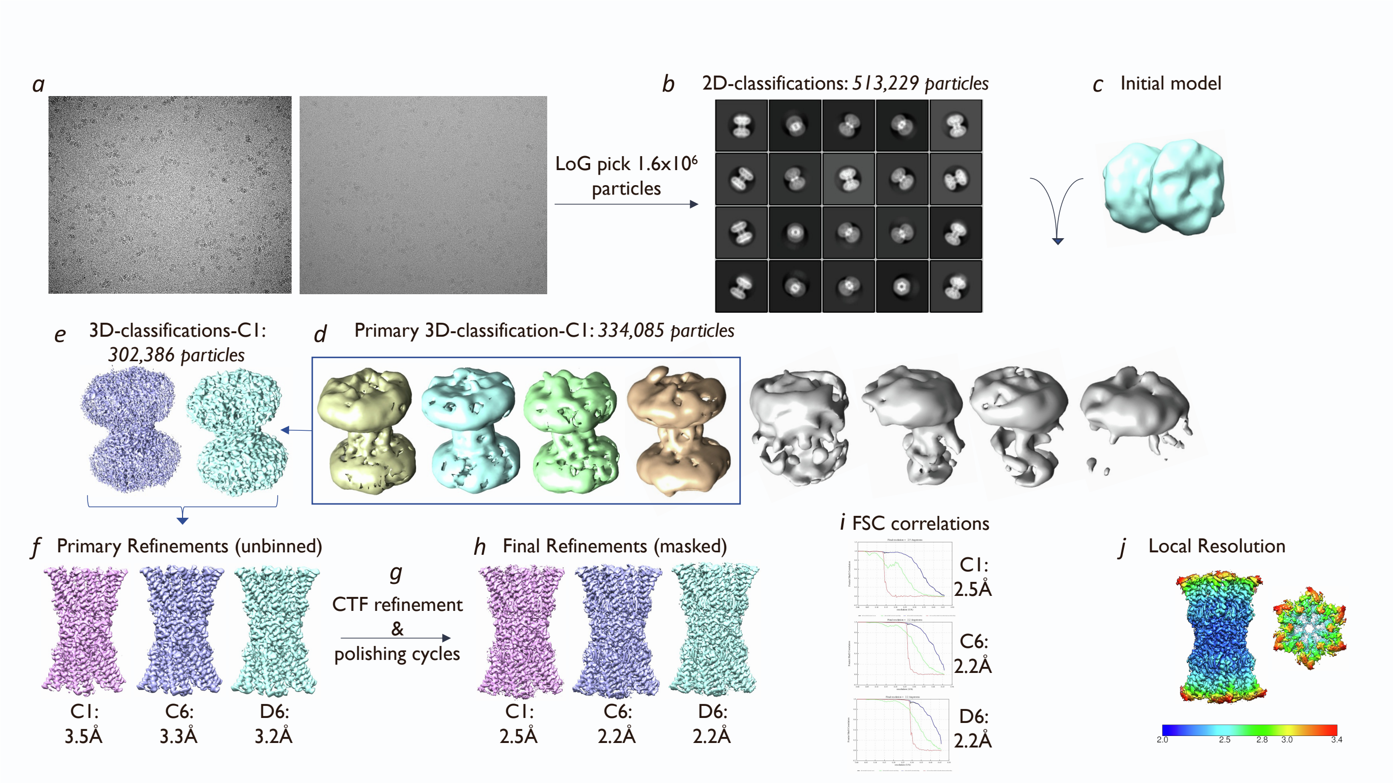

Figure S1: Workflow for processing of cryo-EM data associated with 55mmHg PCO_2 Related to Star Methods. The resolution of reconstructions using C1, C6 and D6 symmetry is shown before and after multiple rounds of particle polishing and CTF refinement. The local resolution map is coloured according to resolution estimated in ResMap (Kucukelbir et al., 2014).

a

Buffer	αCSF20	αCSF55	αCSF90
$\%CO_2$	2.5	10	15
pH pre-gas	7.9	8.7	8.8
pH	7.5	7.5	7.4

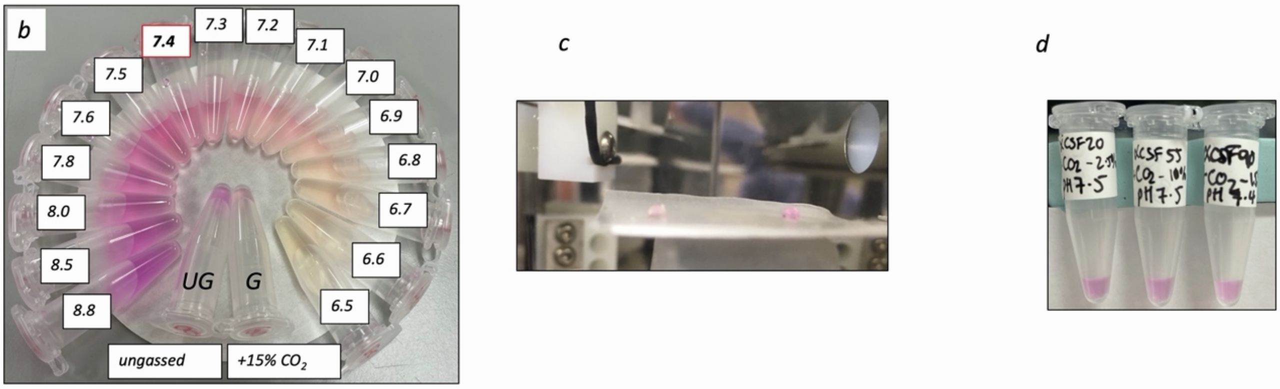

Figure S2: **Control of pH of samples before vitrification.** Related to Star Methods. Controls were carried out to ensure that the pH of the samples at vitrification was the same irrespective of the PCO_2 level. Gassing of the sample with CO_2 is required to obtain the necessary level of PCO_2. **a)** 60 μl samples of each buffer before and after gassing with the indicated percentage of CO_2 showing that all three buffers have the same pH after gassing. The pH was measured using an ultra-microprobe pH meter. **b-d)** To ensure that the pH of the protein remained constant during the blotting procedure samples were tested using Phenol Red as a pH indicator. **b)** To obtain a colour reference for the pH indicator dye, 2μl of Phenol Red was added to 400μl samples of the 90mmHg buffer buffer that had been adjusted to the required pH. The samples prepared as in (a) with 15% CO_2 are shown at the bottom of the wheel (UG not gassed, G gassed). **c)** 3 μl drops of buffer gassed as for protein preparation pre-vitrification (left) and ungassed buffer (right) in the plunge freezer chamber after 30 seconds in the CO_2 - equilibrated atmosphere as described in the methods. The respective colours of the drops do not change over the 30-second period indicating that the pH remains stable during this time. 30 seconds was chosen as being representative of the maximum time taken between applying sample to the grid, and the grid entering the liquid ethane. **d)** The three gassed buffers from (a) with Phenol Red have the same colour.

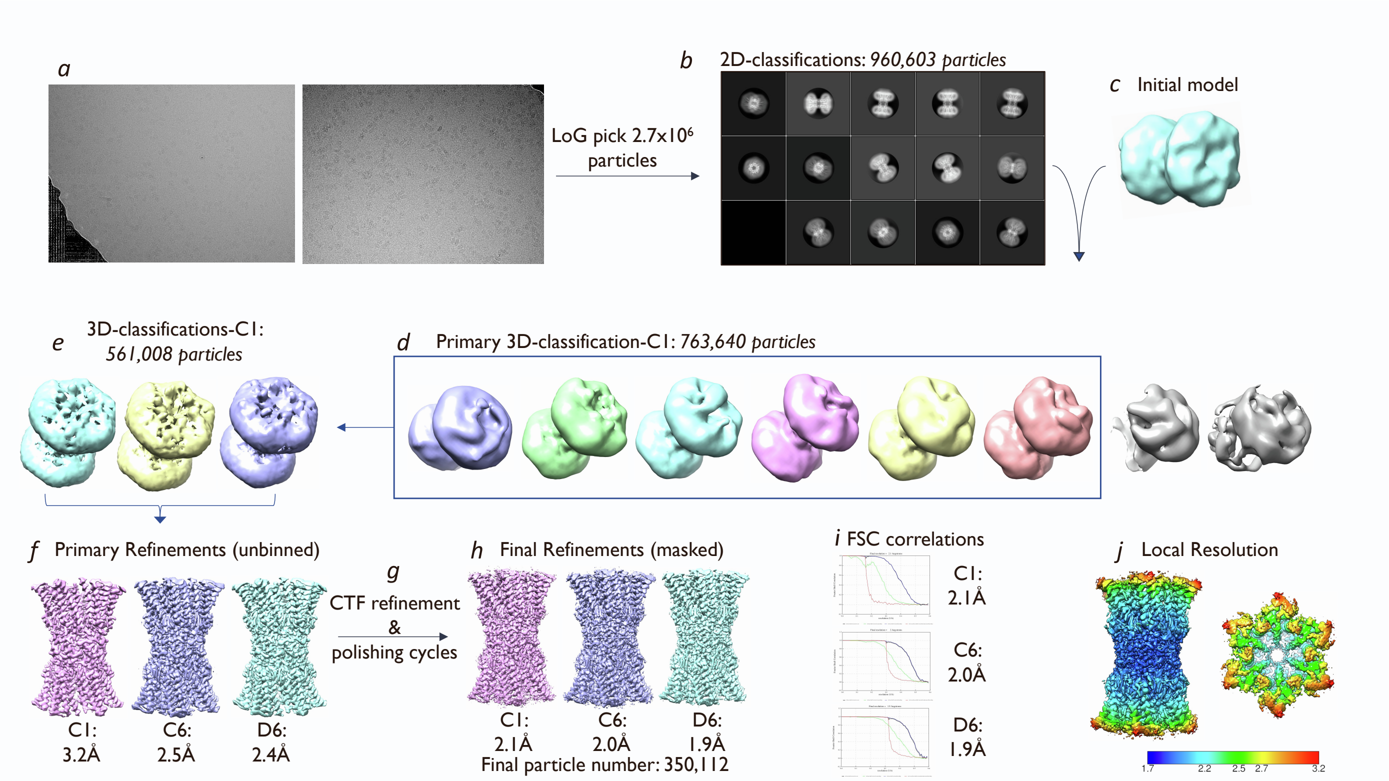

Figure S3: Workflow for processing of cryo-EM data associated with 90mmHg PCO_2. Related to Star Methods. As for Fig. S1 the resolution of reconstructions using C1, C6 and D6 symmetry is shown before and after multiple rounds of particle polishing and CTF refinement. The local resolution map is coloured according to resolution estimated in ResMap (Kucukelbir et al., 2014).

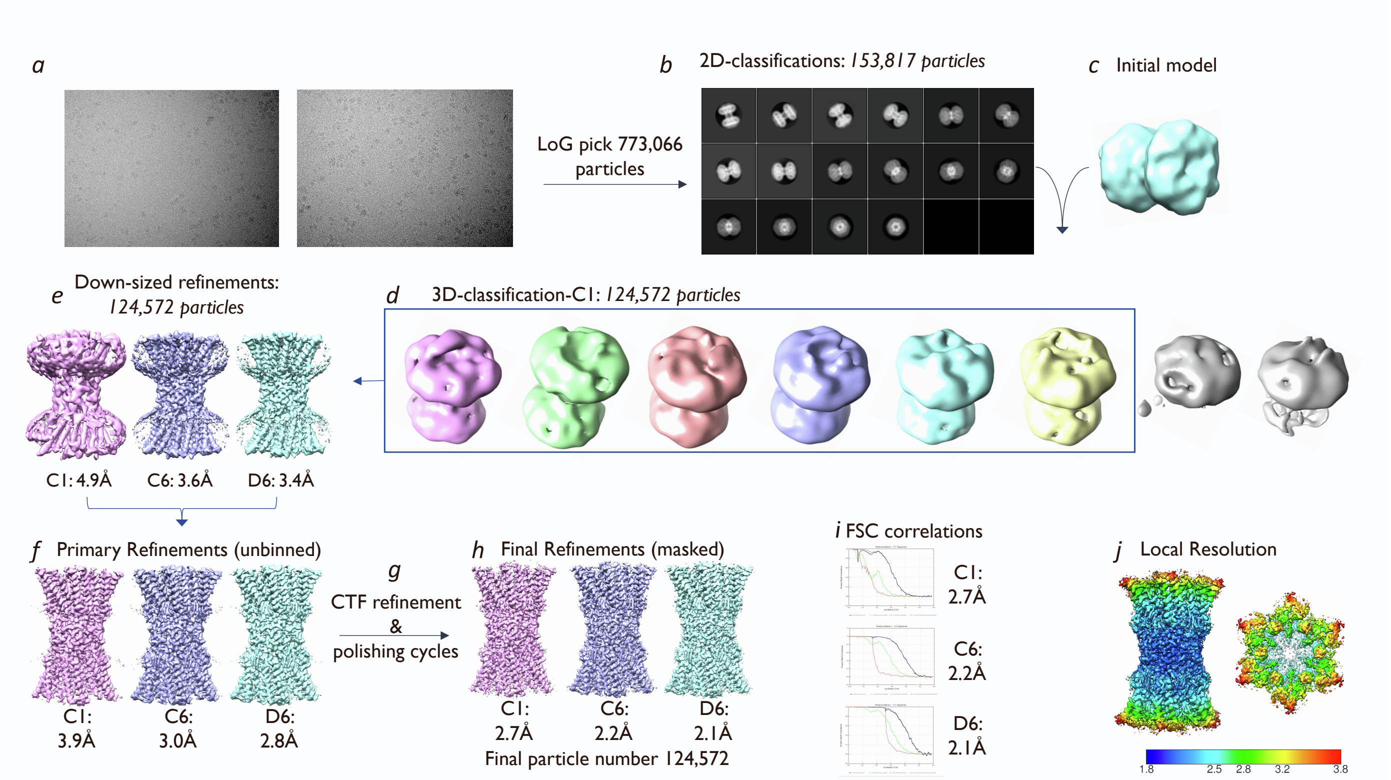

Figure S4: Workflow for processing of cryo-EM data associated with 20mmHg PCO_2. Related to Star Methods. As for Fig. S1 the resolution of reconstructions using C1, C6 and D6 symmetry is shown before and after multiple rounds of particle polishing and CTF refinement. The local resolution map is coloured according to resolution estimated in ResMap (Kucukelbir et al., 2014).

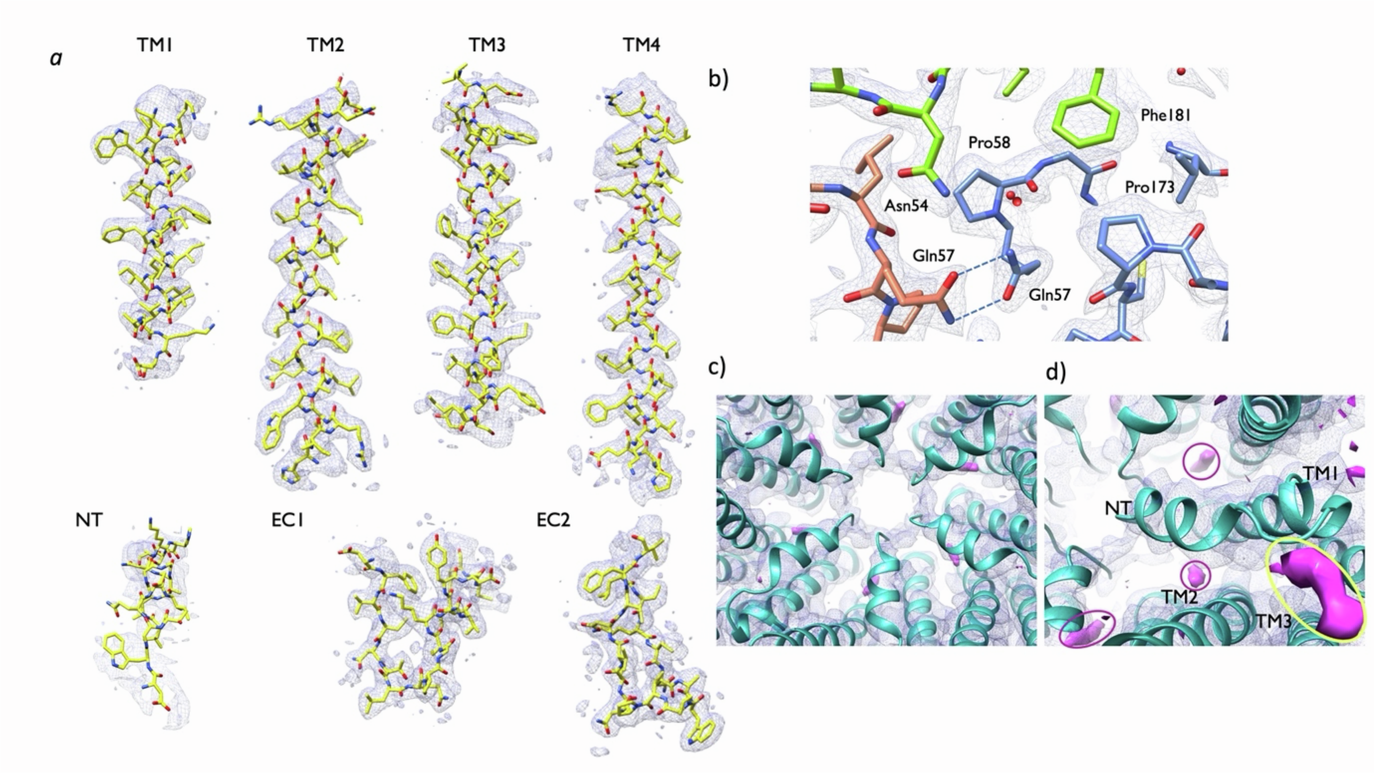

Figure S5: Density for 55 mmHg PCO_2 maps. Related to Star Methods. **a)** Density associated with main structural features (Transmembrane (TM) helices 1-4, the N-terminal helix (NT) and Extracellular (EC) loops 1 and 2.) No sharpening has been applied. (The N-terminus has been shown with a lower threshold (0.0117 *vs* 0.0126).) **b)** Density associated with the gap junction with a map sharpened in Relion (Sharpening B - 22Å^2). **c)** The density associated with the N-terminus can be seen to form a ring in the centre of the pore. **d)** Sausage-shaped densities were observed within the pore (solid magenta circled in magenta) as was density extending from TM3 to the N-terminal helix (solid magenta circled in yellow).

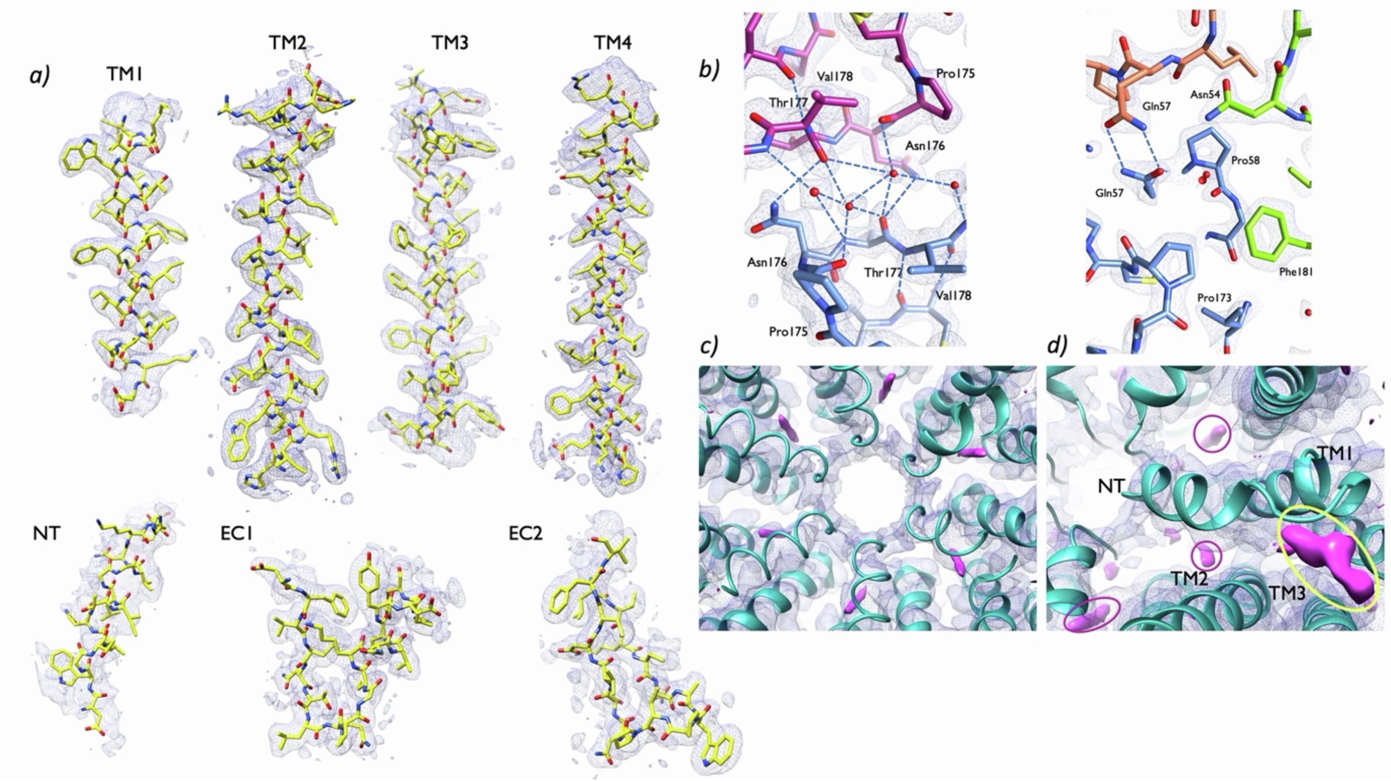

Figure S6: Density for 90mmHg PCO_2 maps. Related to Star Methods. Equivalent to Fig. S2 **a)** Density associated with main structural features (Transmembrane (TM) helices 1-4, the N-terminal helix (NT) and Extracellular (EC) loops 1 and 2.) No sharpening has been applied. (The N-terminus has been shown with a lower threshold (0.0065 *vs* 0.0069).) **b)** Density associated with the gap junction with a map sharpened in phenix.autosharp (Adams et al., 2010). **c)** The density associated with the N-terminus can be seen to form a ring in the centre of the pore. **d)** Sausage-shaped densities were observed within the pore (solid magenta circled in magenta) as was density extending from TM3 to the N-terminal helix (solid magenta circled in yellow).

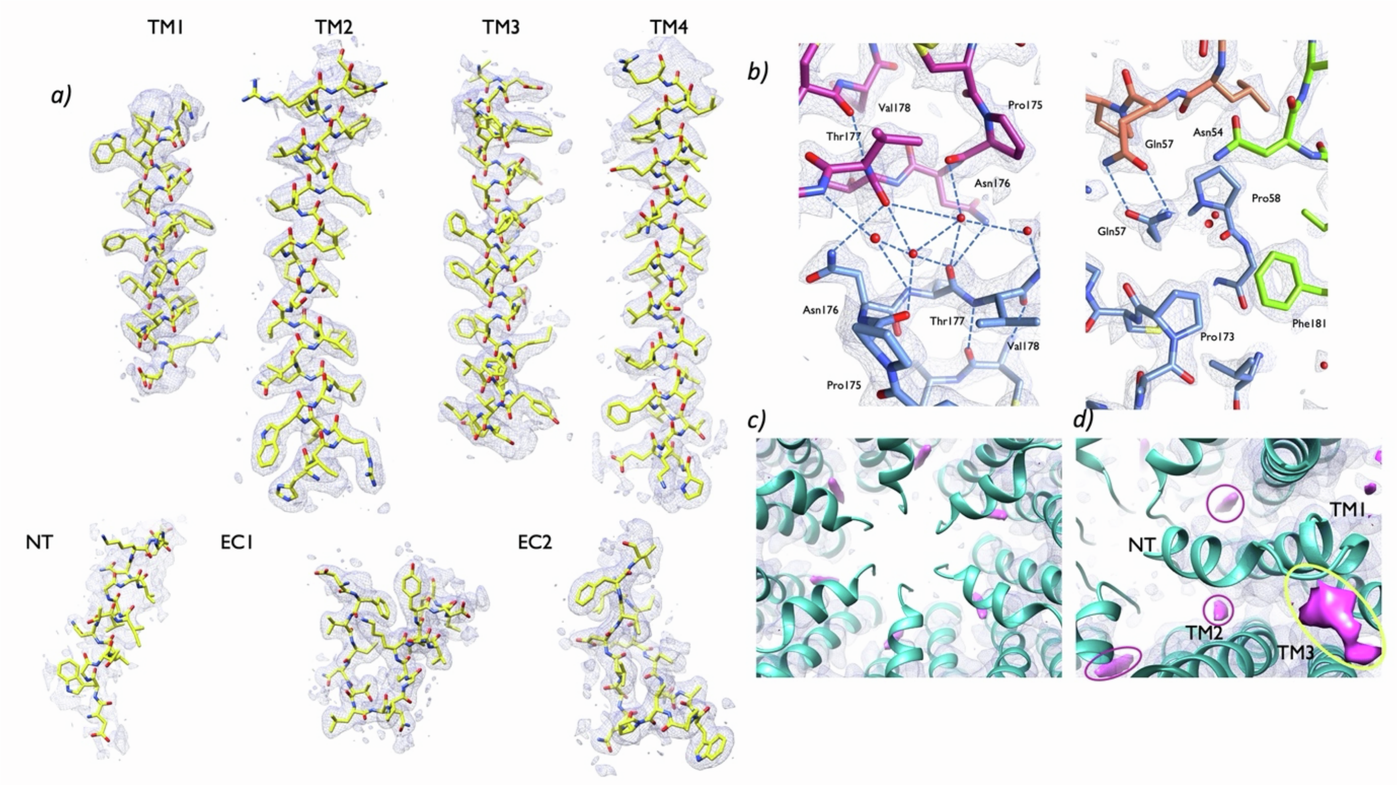

Figure S7: Density for 20mmHg PCO_2 maps. Related to Star Methods. Equivalent to Fig. S2 **a)** Density associated with main structural features (Transmembrane (TM) helices 1-4, the N-terminal helix (NT) and Extracellular (EC) loops 1 and 2.) No sharpening has been applied. (The N-terminus has been shown with a lower threshold (0.0065 *vs* 0.0069).) **b)** Density associated with the gap junction with a map sharpened in phenix.autosharp (Adams et al., 2010). **c)** The density associated with the N-terminus is much less defined than in Figs S5 and S6. **d)** Sausage-shaped densities were observed within the pore (solid magenta circled in magenta) as was density extending from TM3 to the N-terminal helix (solid magenta circled in yellow).

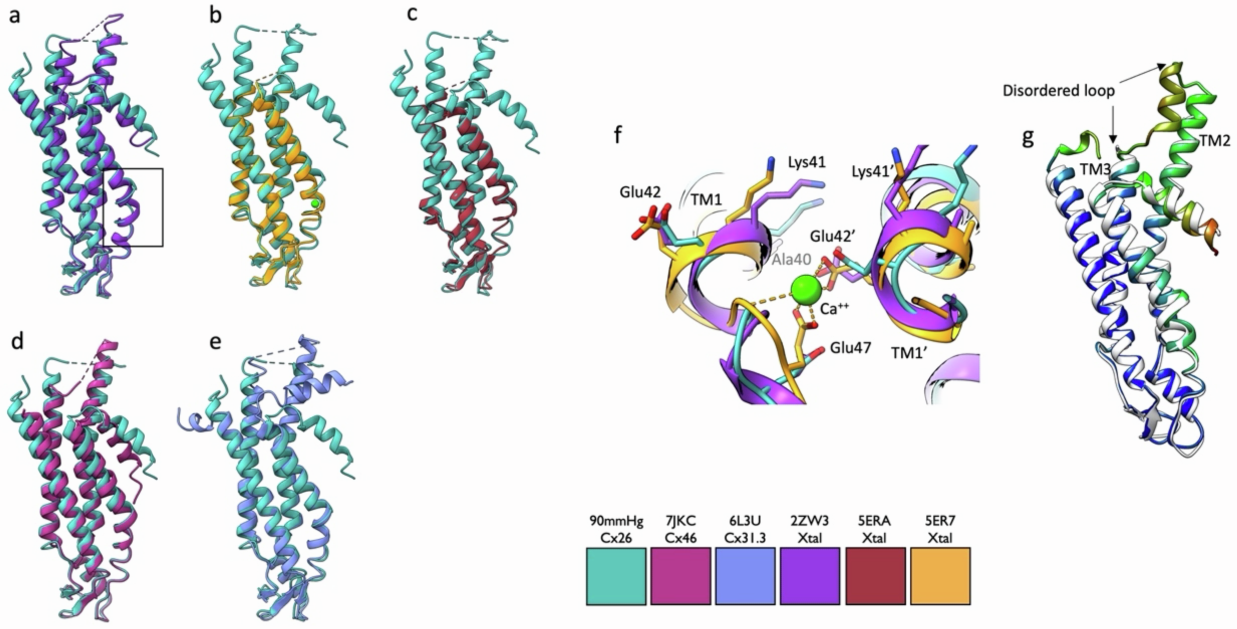

Figure S8: Comparison of structures of connexins. Related to Figure 3. Structure of a subunit of 90mmHg PCO_2 Cx26 cryo-EM structure (sea-green) superposed on: **a)** Cx26 crystallised in UDM (Maeda et al., 2009) (2ZW3, purple); **b)** Cx26 calcium-bound, crystallised in a facial amphiphile (Bennett et al., 2016) (5ER7, orange); **c)** Cx26 crystallised as in (b) but without calcium (5ERA, brown); **d)** cryo-EM structure of Cx46 (Flores et al., 2020) (7JKC, burgundy) and **e)** cryo-EM structure of Cx31.3 (Lee et al., 2020) (6L3U, sky blue). The N-termini are not observed in either 5ER7 or 5ERA and are in a raised position in 6L3U. The region between EC1 and TM1 (boxed in (a), residues Val 37 to Glu 42) varies amongst the Cx26 structures. Using the refined cryo-EM structure as a search model for molecular replacement against structure factor amplitudes associated with either the 5ER7 or 5ERA models clearly shows the resulting density to be consistent with the crystallographic models. **f)** Comparison of the cryo-EM structure with 2ZW3 and 5ER7 with the same colouring scheme as in a-e. Residues between Val37 and Glu42 vary amongst all the structures; residues Glu42 to Glu47 between the calcium-bound and non-bound structures. **g)** Structure of Cx26 cryo-EM structure (90mmHg PCO_2 white) superposed on a model predicted by AlphaFold (Jumper et al., 2021). The model from AlphaFold is coloured by confidence level with low (red) to high (blue) confidence spanning the colours of the rainbow. The boundaries of the loop that is not seen in any of the crystal structures is delimited by arrows. Both AlphaFold and RosettaFold (Baek et al., 2021) consistently predict a similar conformation, though with low confidence levels.

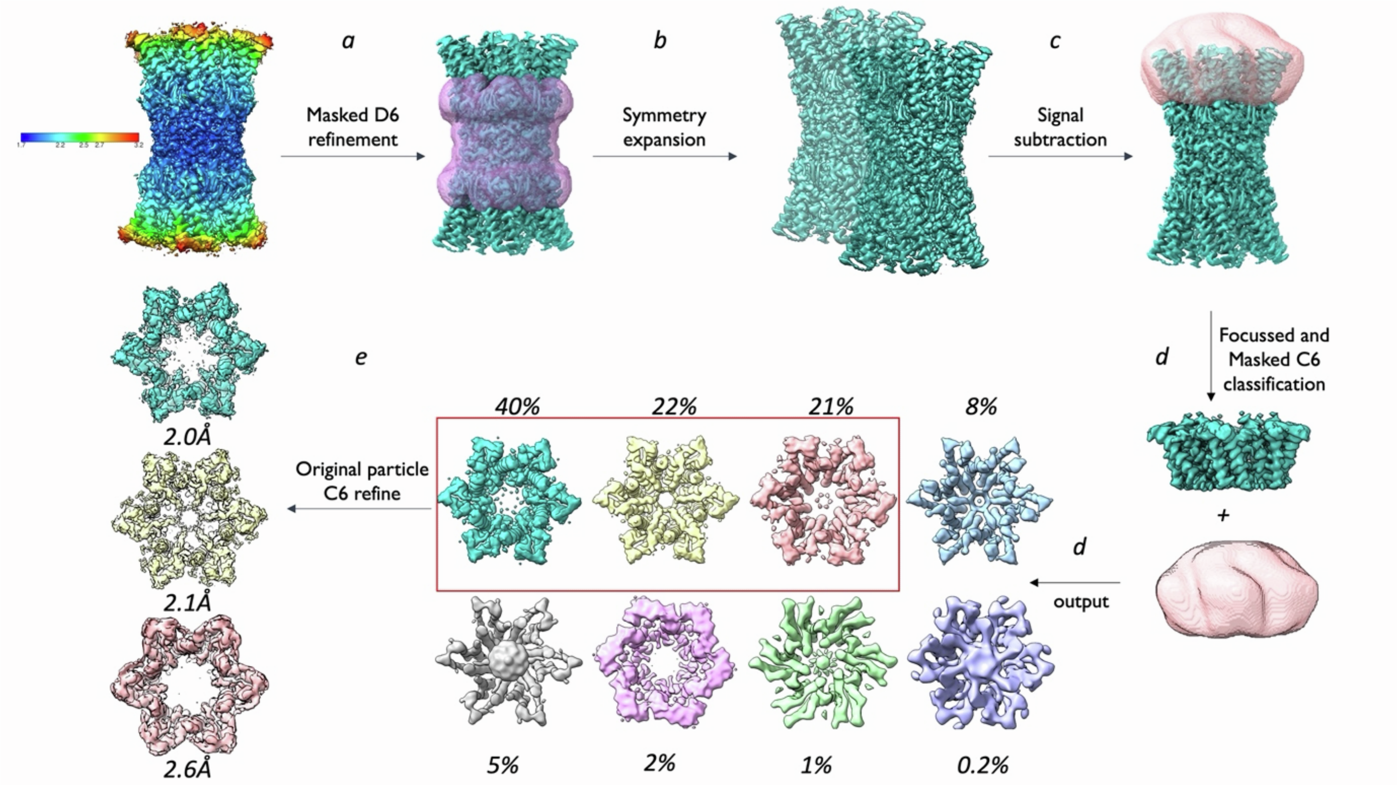

Figure S9: C6 classification of the 90mmHg D6 refined particles. Related to Star Methods. **a)** the best refined D6 particles were taken, masked around the region nearer to the hemichannel docking site, and refined further **b)** these particles were symmetry expanded in D1 **c)** the symmetry expanded particle set were signal subtracted using a second mask covering the least-defined area. **d)** these particles were centred, and classified imposing C6 symmetry, fixed angles, and 8 classes. **e)** the top three classes were selected, and the original particles refined with C6 symmetry imposed, using a mask covering one hemichannel. The colours of the reconstructions after refinement match those in the red box.

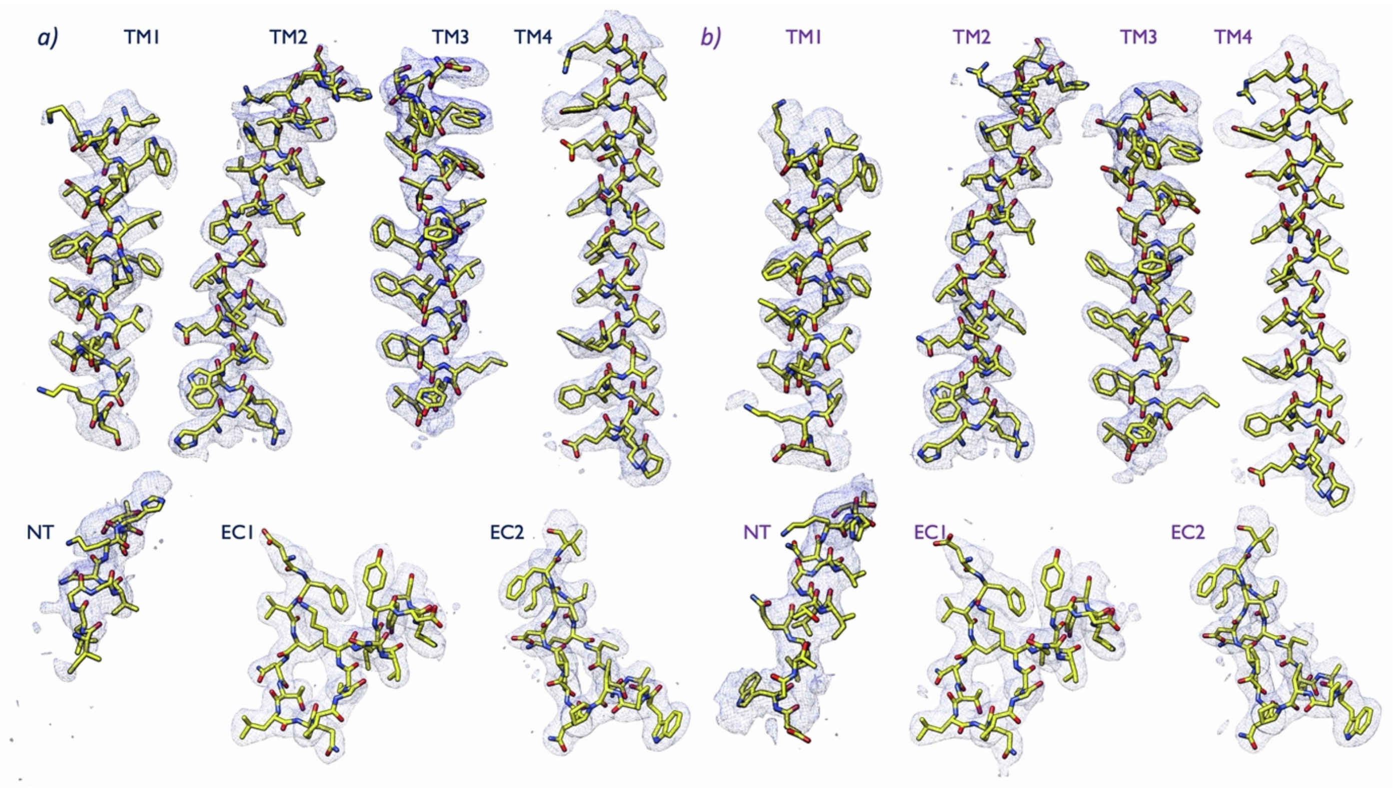

Figure S10: Quality of density in maps resulting from C6 classifications in Fig S9. Related to Star Methods. **a)** Density associated with main structural features for the refined particles in class 1. No sharpening has been applied (threshold (0.0075). **b)** Density associated with main structural features for the refined particles in class 2. No sharpening has been applied (threshold (0.0075)).

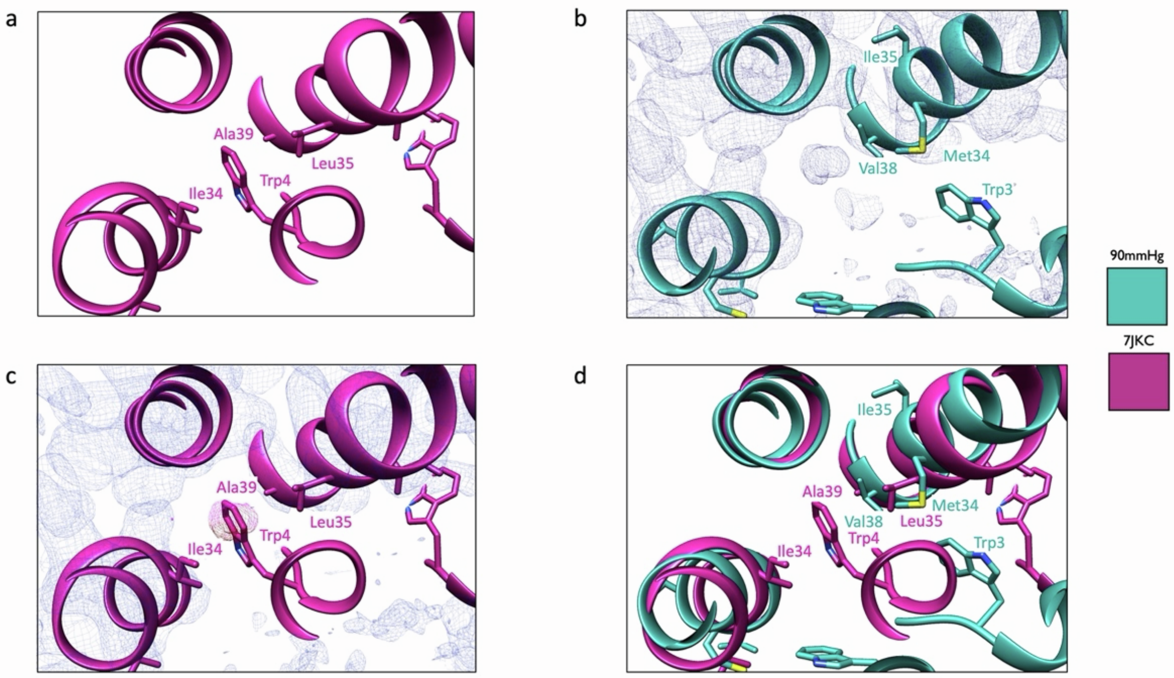

Figure S11: The interaction of the N-terminus with TM1 in Cx26 and Cx46. Related to Star Methods and Figure 3. **a)** Cx46 (7JKC) showing Trp4 seated between Ala39 and Leu35 of TM1 on one subunit and Ile34 on TM1 of the adjoining subunit. **b)** A similar view for the 90mmHg PCO_2 structure with the associated density. **c)** Density as in b) with Cx46 structure as in a) shows the overlap between Trp4 of Cx46 and the lipid like density (pink) associated with the Cx26 structures. **d)** overlay of Cx46 and Cx26, showing the difference in position of the N-terminal Trp.

	55 mmHg CO_2	90 mmHg CO_2	20 mmHg CO_2
Voltage (kV)	300	300	300
Magnification (x1000) X	81	105	105
Camera	K3	K3	K3
Camera mode	Super-resolution	Super-resolution	Super-resolution
Energy filter (eV)	20	20	20
Defocus range (mm)	-2 to -1	-2 to -1	-2 to -1
Pixel size (Å/pix)	1.09	0.85	0.85
Dose on detector (e^-/pix/sec)	10	10	10
Dose on sample (e^-/pix/sec)	10.9	11	11
Exposure time	5	3	3
No. of images	4003	11647	3305
Frames per image	45	45	45
Initial particle number	1679668	2655822	773121
Final particle number	344085	360112	124572
**Resolution**[a]			
masked D6	2.2	1.9	2.1
masked C6	2.2	2.0	2.2
masked C1	2.5	2.1	2.7

a) From Relion_postprocess (Zivanov et al., 2018)

Table S1: Cryo-EM data collection and processing statistics, Related to Star Methods

**Refinement**	**55mm Hg CO_2**	**90mm Hg CO_2**	**20mmHg CO_2**
Initial model used (PDB code)	2ZW3	55mm Hg CO_2	55mm Hg CO_2
Resolution (Å; FSC=0.5) Map	2.2	1.9	2.1
Sharpening B factor ($Å^2$)	0	0	0
**Model composition**	12 subunits	12 subunits	12 subunits
Non-hydrogen atoms	19097	19313	19205
Protein residues	2244	2268	2256
Water	305	305	305
Ligand: lipid/detergent	60	60	60
**B factor ($Å^2$)**			
protein	71	63	72
water	42	36	42
Lipid/detergent	65	57	64
**R.m.s. deviations**			
Bond lengths (Å)	0.008	0.002	0.004
Bond angles (°)	0.615	0.479	0.501
**Validation**			
MolProbity score	1.45	1.05	1.29
Clashscore	3.04	2.19	1.99
Rotamer outliers (%)	2.94	1.18	2.38
**Ramachandran plot**			
Favoured (%)	98.36	98.92	97.83
Allowed (%)	1.64	1.08	2.17
Disallowed (%)	0.0	0.0	0.0
**Map resolution (Å)**	2.2	1.9	2.1
FSC threshold	0.143	0.143	0.143
Map resolution range (Å) (unmasked)	2.1/2.2/2.2	1.8/1.8/2.0	1.9/1.9/2.2
Map resolution range (Å) (masked)	2.1/2.1/2.2	1.7/1.8/1.9	1.9/1.9/2.1
**Correlation coefficients**			
CC (mask)	0.88	0.90	0.90
CC (box)	0.84	0.82	0.82
CC (peaks)	0.76	0.78	0.76
CC (volume)	0.87	0.89	0.89
Mean CC for ligands	0.80	0.85	0.80

Table S2: Cryo-EM refinement and validation statistics, Related to Star Methods and Figure 1

	Class 1	Class 2
Initial model used	90mmHg CO_2	90mmHg CO_2
Resolution (Å)	2.0	2.1
Sharpening B factor ($Å^2$)	0	0
Model composition	6 subunits	6 subunits
Non-hydrogen atoms	9483	9756
Protein residues	1092	1134
Water	297	234
Ligand: lipid/detergent	30	30
**B factor ($Å^2$)**		
protein	64	73
water	46	48
Lipid/detergent	62	68
**R.m.s. deviations**		
Bond lengths (Å)	0.011	0.011
Bond angles (°)	0.825	0.864
**Validation**		
MolProbity score	2.33	2.65
Clashscore	5.68	8.31
Rotamer outliers (%)	8.59	13.71
**Ramachandran plot**		
Favoured (%)	95.51	95.14
Allowed (%)	3.93	4.32
Disallowed (%)	0.56	0.54
**Map resolution (Å)**	2.1	2.2
FSC threshold	0.143	0.143
Map resolution range (Å) (unmasked)	1.8/1.8/2.0	1.9/2.0/2.2
Map resolution range (Å) (masked)	1.7/1.8/2.0	1.9/1.9/2.1
**Correlation coefficients**		
CC (mask)	0.91	0.9
CC (box)	0.78	0.79
CC (peaks)	0.72	0.72
CC (volume)	0.89	0.89

Table S3: Refinement and Validation Statistics 90mmHg PCO_2 hemichannel models, Related to Star Methods and Figure 4.
